# Comparison of the bacterial microbiome in the pharynx and nasal cavity of persistent, intermittent carriers and non-carriers of Staphylococcus aureus

**DOI:** 10.1099/jmm.0.001940

**Published:** 2024-12-04

**Authors:** Samuel González-García, Aida Hamdan-Partida, Julia Pérez-Ramos, José Félix Aguirre-Garrido, Anaíd Bustos-Hamdan, Jaime Bustos-Martínez

**Affiliations:** 1 Doctorado en Ciencias Biológicas y de la Salud, Universidad Autónoma Metropolitana, Mexico City, Mexico; 2 Departamento de Atención a la Salud, UAM Xochimilco, Calzada del Hueso 1100, Colonia Villa Quietud, Alcaldía Coyoacán, C.P. 04960, CDMX, Mexico; 3 Departamento de Sistemas Biológicos, UAM Xochimilco, Calzada del Hueso 1100, Colonia Villa Quietud, Alcaldía Coyoacán, C.P. 04960, CDMX, Mexico; 4 Departamento de Ciencias Ambientales, UAM Lerma, Av. de las Garzas 10E, l Panteón 52005, Municipio Lerma de Villada, Estado de México, Mexico

**Keywords:** carriers, metagenomic comparison, non-carriers, nose, pharynx, *Staphylococcus aureus*

## Abstract

**Introduction.***Staphylococcus aureus* is a bacterium that colonizes various human sites. The pharynx has been considered as a site of little clinical relevance and little studied. Recently, it has been reported that *S. aureus* can colonize more the pharynx than the nose. In addition, *S. aureus* can persist in these sites for prolonged periods of time.

**Hypothesis.** The composition of the pharyngeal and nasal microbiome will differ between persistent, intermittent carriers and non-carriers of *S. aureus*.

**Aim.** Determine whether the pharyngeal and nasal microbiome is different between carriers and non-carriers of *S. aureus*.

**Methodology.***S. aureus* carriers were monitored by means of pharyngeal and nasal exudates of apparently healthy adult university students for 3 months. Samples from individuals of the same carrier type were pooled, and DNA was extracted and the 16S rRNA was sequenced. The sequences were analysed in MOTHUR v.1.48.0 software, by analysing the percentages of relative abundance in the STAMP 2.1.3 program, in addition to the predictive analysis of metabolic pathways in PICRUSt2.

**Results.** A greater colonization of *S. aureus* was found in the pharynx than in the nose. The microbiomes of *S. aureus* carriers and non-carriers do not show significant differences. The main microbiome difference found was between pharyngeal and nasal microbiomes. No significant differences were found in the abundance of the genus *Staphylococcus* in pharyngeal and nasal *S. aureus* carriers and non-carriers. The nasal microbiome was found to have more variation compared to the pharyngeal microbiome, which appears to be more stable between individuals and pools. Predictive analysis of metabolic pathways showed a greater presence of *Staphylococcus*-associated pathways in the nose than in the pharynx.

**Conclusion.***S. aureus* can colonize and persist in the pharynx in equal or greater proportion than in the nose. No statistically significant differences were found in the microbiome of the pharyngeal and nasal carriers and non-carriers of *S. aureus*, but the pharyngeal and nasal microbiomes are different independent of the type of *S. aureus* carrier or non-carrier. Therefore, the microbiome apparently does not influence the persistence of *S. aureus*.

**Impact Statement**

This is the first study on the difference in the pharyngeal and nasal microbiome between *Staphylococcus aureus* carriers and non-carriers. This is of interest since it is not known whether the microbiome plays a role in the persistence of *S. aureus*. We found that there is a difference between the nasal and pharyngeal microbiomes; however, this difference apparently does not influence the persistence of *S. aureus*. In addition, raw DNA sequences of metagenomic pools obtained at the end of the follow-up of *S. aureus* carriers and non-carriers in the pharynx and nose were analysed and stored at the NCBI, which will allow other scientists to reuse the data obtained in this work.

## Data Summary

All raw sequence is available in the NCBI in the BioProject PRJNA1051577:

https://dataview.ncbi.nlm.nih.gov/object/PRJNA1051577?reviewer=4hsqoub9kojfbch0d5b2h60csp.

## Introduction

The human body offers a great diversity of niches for specific microbiomes, variable consortia and/or bacterial communities [1]. A wide diversity of micro-organisms, including archaea, bacteria, fungi, viruses and protozoa, coexist in the pharyngeal and nasal cavities of humans, which have been reported in laboratory cultures [2,3], performing important functions such as inhibiting infections by pathogens, regulating metabolism and stimulating the host immune system [4,5].

The nasal and oral cavities, including the pharynx, are the major sites of entry into the human body for micro-organisms, which can colonize the oral environment and from there spread through the epithelia to other sites in the body such as the trachea, lungs, stomach and intestines [4]. The pharynx, as an ecological niche, is an important environment for many commensal bacteria, as well as for some potentially invasive pathogens such as *Haemophilus influenzae*, *Streptococcus pneumoniae*, *Neisseria meningitidis* and *Staphylococcus aureus* [6,8]. The bacterial communities of the pharynx constitute a microenvironment in the surroundings of the epithelial cells, with important interactions between them; however, knowledge of the composition and prevalence of the microbiota in the pharynx is still insufficient [9].

The Human Microbiome Project (HMP) identified five predominant bacterial phyla in the pharynx: *Bacillota* (also called *Firmicutes*), *Bacteroidetes*, *Proteobacteria*, *Actinobacteria* and *Fusobacteria*. The microbiome of the pharynx is distinct from the microbiome of other parts of the body such as the gut, skin and vaginal cavity, one difference being the higher abundance of *Bacteroidetes* in the pharynx compared to the other sites mentioned. The phyla *Bacteroidetes* and *Proteobacteria* are important in human infections since several pathogenic bacteria are found here [2]. For the pharynx to resist infection by native species such as *Streptococcus* and *Haemophilus*, it is necessary to maintain homeostasis of the microbiome. Several species of pathogens such as *S. aureus*, *H. influenzae*, *Mycoplasma pneumoniae* and *S. pneumoniae* survive and persist in the pharyngeal environment and can colonize, without causing an infection [2,10].

Besides, the nose is an important cavity in contact with the external environment. When breathing, the respiratory system is exposed to aeroallergens, pollutants, fungal spores and micro-organisms [11]. The nose is colonized by a great diversity of bacteria (both pathogenic, commensal and opportunistic) [12,14]. This diversity in the nature of the microbiome is attributed to niche-specific factors such as their position in the respiratory tract or temperature and humidity [11]. The microbiome of the entire upper respiratory tract, including the nasal tract, is almost always composed of the same microbial communities [15].

The nasal microbiome evolves very rapidly from birth to early childhood. Some commensal bacteria have been associated with a healthy nasal microbiome, as they play important roles in protecting the respiratory tract (e.g. *Corynebacterium*, *Dolosigranulum* and *Staphylococcus lugdunensis*) [16,21].

*S. aureus*, *H. influenzae* and *S. pneumoniae* are opportunistic nasal and pharyngeal pathogens and can cause a variety of diseases if they colonize susceptible tissue or if the host is immune deficient [22,25]. Therefore, the microbiome of the nasal passages of healthy adults has been observed to be dominated by three phyla: *Actinobacteria*, *Bacillota* and *Proteobacteria* [11,12, 26, 27]. However, the distinction between who is a pathogenic and commensal bacterium may not be obvious, as some bacteria, such as *S. aureus*, have the capacity to be versatile commensal opportunists, causing significant morbidity and mortality [28,31]. Instead, various environmental factors have been reported to modify the composition of microbiomes [32,33].

In this regard, *S. aureus* colonizes approximately 30% of the world’s population, with the nose being the most studied site of colonization (20–80%) [34,35]. Its prevalence has been reported in other anatomical sites such as the abdomen (15%), intestines (17–31%), perineum (22%), vagina (22%), axillae (8%) and especially the pharynx (4–64%), with the latter showing the greatest variability in reports [6,36,42].

Although *S. aureus* can colonize a wide variety of tissues by participating as part of the microbiota [43], it is still responsible for a variety of community and nosocomial infections [44] and can also cause infective endocarditis and bacteremia, as well as pleuropulmonary, skin and soft tissue infections and even contamination of medical devices [45]. In the case of invasive *S. aureus* infections, they are associated with a mortality rate greater than or equal to 20% [46,47].

Several studies show that *S. aureus* can not only colonize but also persist in the human nose and pharynx [38,48, 49], following three patterns of carriers in the population: persistent carriers (always present *S. aureus* when samples are taken), intermittent carriers (not always present *S. aureus* when samples are taken, sometimes it is present and sometimes not) and non-carriers (never present *S. aureus* in the samples taken) [50]. It has been shown that persistent carriers tend to have only one clone at the time of sampling. They also favour the dissemination of the bacterium to the environment and are more likely to be reinfected compared to intermittent carriers and non-carriers. In the case of intermittent carriers, they may have different strains over time and less colonization [6,51].

Although there are studies of differences in the nasal and pharyngeal microbiome composition in healthy people [52] or in those with diseases such as asthma [53] and allergic rhinitis [54] or in smokers [55], there are no reports of the microbiome composition in asymptomatic carriers and non-carriers of *S. aureus* in the pharynx and nose. Therefore, the aim of this work is to compare the composition of the pharyngeal and nasal microbiome of apparently healthy individuals who are persistent carriers, intermittent carriers or non-carriers of *S. aureus*.

## Methods

### Sample collection

Pharyngeal and nasal swabs were collected from 98 apparently healthy students from a public university in Mexico City; 65 were female (66.3%) and 33 male (33.7%), with a mean age of 21.1 years (±3.3), who were asked to sign a letter of informed consent and privacy of personal data. Swabs were taken serially once a month for 3 months, without antibiotic treatment for at least 8 days before. Swabs of exudates were incubated in trypticasein soy broth (TSB) (Bioxon, Mexico) for 24 h, followed by seeding on salt and mannitol agar (Becton Dickinson, Mexico) [56]. In the last sampling, a second pharyngeal and nasal exudate was collected and stored in microcentrifuge tubes with 1 ml of sterile PBS 1× solution at −70 °C.

### *S. aureus* carrier type

Samples stored in TSB were seeded on salt and mannitol agar, identifying all isolates that were positive to mannitol and coagulase tests as *S. aureus* [56]. If the coagulase test was doubtful, the 16S rRNA gene was sequenced to confirm the presence of *S. aureus*.

For the identification of *S. aureus* carrier type, nine carrier groups were considered: (1) persistent in the pharynx and nose (person who always had isolates in both sites); (2) persistent exclusively in the pharynx (person who always had isolates in the pharynx and no isolates in the nose); (3) persistent exclusively in the nose (person who always had isolates in the nose and no isolates in the pharynx); (4) persistent in the nose and intermittent in the pharynx (person who always had isolates in the nose and does not always present *S. aureus* in the pharynx); (5) persistent in the pharynx and intermittent in the nose (person who has always had isolates in the pharynx and does not always present *S. aureus* in the nose); (6) intermittent in the pharynx and nose (person who does not always present *S. aureus* in the pharynx and nose); (7) pharynx-only intermittent (person who does not always present *S. aureus* in the pharynx and never presented *S. aureus* in the nose); (8) nose-only intermittent (person who does not always present *S. aureus* in the nose and never presented *S. aureus* in the pharynx); and (9) non-carriers (person who never had *S. aureus* in the pharynx and nose) (Table 1).

**Table 1.** Types of pharyngeal and nasal carriers of *S. aureus*

*S. aureus* carrier type	no. of carriers	Percentage
1. Persistent P and N	18	18.3
2. Exclusive Persistent P3. Exclusive Persistent N	190	19.30
4. Intermittent P and N	15	15.3
5. Persistent N and intermittent P	5	5.2
6. Persistent P and intermittent N	15	15.3
7. Intermittent exclusive P	20	20.4
8. Intermittent exclusive N	0	0
9. No carriers	6	6.2

NnosePpharynx

#### Detection of methicillin-resistant *S. aureus* (MRSA) strains

For the identification of MRSA strains, the oxacillin MIC test was performed following the CLSI methodology [57]. Strains that grew at a concentration ≥4 µg ml^−1^ were considered MRSA, using the *S. aureus* ATCC 43300 strain as a positive control and the ATCC 29213 strain as a negative control.

### DNA extraction and sequencing of the V3–V4 region of the 16S rRNA gene

For the extraction of total DNA from carrier samples, sample pools (SPs) were performed according to the number of carriers obtained for each type of *S. aureus* carrier, trying to have at least three pools and that each pool had at least one sample, so the number of samples per pool depended on the total number of samples of each type. Therefore, the number of samples per pool depended on the total number of samples of each type, according to the data shown in Table S1 (available in the online version of this article), which indicates the pools for each type of carrier. The samples were divided into the following 6 groups as shown in Table 2: (i) persistent in the pharynx (P-Persist), which included 52 pharyngeal samples from carrier groups 1, 2 and 6 (Table 2) ; (ii) persistent in the nose (N-Persist), which included 23 nasal samples from carrier groups 1, 3 and 5 (Table 1); (iii) pharyngeal intermittent (P-Int), including 40 pharyngeal samples from carrier groups 4, 5 and 7 (Table 1); (iv) nasal intermittent (N-Int), including 30 nasal samples from carrier groups 4 and 6 (Table 1); (v) pharyngeal non-carriers (P-NC), including 6 pharyngeal samples from carrier group 9; and (vi) nasal non-carriers (N-NC), including 6 nasal samples from carrier group 9 (Table 2).

**Table 2.** *S. aureus* carrier and non-carrier groups and alpha-diversity analysis

*S. aureus* carrier group	Pools	OTUs	Coverage	Inv Simpson (lci-hci)	Shannon (lci-hci)	Chao (lci-hci)
P-Persist (*n*=52), 9 SPs	1 SP of 7 samples5 SPs of 6 samples3 SPs of 5 samples	188	0.98	12.02 (11.59–12.49)	2.96 (2.92–3.00)	590.31 (406.83–930.41)
P-Int (*n*=40), 9 SPs	2 SPs of 7 samples1 SP of 6 samples3 SPs of 5 samples2 SPs of 2 samples1 SP of 1 sample	142	0.99	9.84 (9.45–10.26)	2.94 (2.90–2.98)	363.18 (247.81–606.93)
P-NC (*n*=6), 3 SPs	3 SPs of 3 samples	193	0.98	11.20 (10.78–11.66)	2.88 (2.84–2.92)	573.58 (401.45–892.35)
N-Persist (*n*=23), 6 SPs	3 SPs of 6 samples2 SPs of 2 samples1 SP of 1 sample	153	0.98	4.39 (4.22–4.56)	1.66 (1.62–1.70)	442.37 (300.83–746.85)
N-Int (*n*=30), 6 SPs	6 SPs of 5 samples	241	0.98	10.95 (10.56–11.36)	2.79 (2.74–2.83)	537.88 (421.74–729.80)
N-NC (*n*=6), 3 SPs	3 SPs of 2 samples	122	0.99	1.75 (1.70–1.80)	1.04 (0.99–1.09)	426.13 (272.48–754.08)

hcihigher bound of confidence intervallcilower bound of confidence intervalOTUsoperational taxonomic unitsSPsSample Pool

For extraction, the tubes with the samples were centrifuged for 10 min at 13 000 r.p.m. to concentrate the cells, and the supernatant was removed and resuspended with 1 ml of another sample of another carrier with the same type of characteristics. The procedure was repeated as many times as necessary to obtain the corresponding number of samples in the pool. The final supernatant was removed, and the pellet was resuspended with 480 µl of 50 mM EDTA solution, adding 60 µl of lysozyme (10 mg ml^−1^) and 5 µl of lysostaphin (5 mg ml^−1^) and incubating for 1 h at 37 °C. Subsequently, the samples were centrifuged for 3 min at 13 000 r.p.m., and the supernatant was discarded.

DNA extraction was performed with the ReliaPrep gDNA Tissue Miniprep System kit (Promega, USA), following the supplier’s instructions for buccal swabs. Subsequently, a 1% agarose gel electrophoresis in 0.5× Tris-Borate-EDTA buffer was performed to confirm the DNA extraction and quantified in a BioPhotometer (Eppendorf, Germany).

DNA extracted from the SP was sent for sequencing in the V3–V4 region of the 16S rRNA gene to Integrated Microbiome Resource (IMR, Canada), using Illumina 1.9 sequencing technology. Table 2 shows the number of SP per carrier group and how many individuals make up each SP.

The Sequence Read Archive (SRA) files of the 36 SPs are publicly available under BioProject PRJNA1051577 in the repository of the National Center for Biotechnology Information (NCBI).

### Bioinformatic processing

Sequences of the V3–V4 region of the 16S rRNA gene were analysed using MOTHUR v.1.48.0 software [58,62], following the MiSeq SOP protocol [63] (https://mothur.org/wiki/miseq_sop/), normalizing the analysis to the file with the lowest number of sequences.

Alpha-diversity analysis was performed with the estimated number of operational taxonomic units (OTUs), obtaining inverse Simpson’s, Shannon’s and Chao’s indices.

### Predicting functional analysis of pharyngeal and nasal microbiomes

The prediction of functional characteristics of bacterial communities was performed by phylogenetic investigation of communities through unobserved state reconstruction (PICRUSt2) [64,65] using the Kyoto Encyclopedia of Genes and Genomes (KEGG, http://www.genome.jp/kegg/) database [66].

### Statistical analysis

For statistical analysis of *S. aureus* carriers and non-carriers, one-way and two-way ANOVA followed by Tuckey’s test was performed in GraphPad Prism software v.8.0.1 for Windows, and for beta-diversity, predictive and statistical analyses of metataxonomic data, STAMP 2.1.3 software was used [67], performing multiple comparison ANOVA followed by the Tukey–Kramer test, considering *P*-values <0.05 statistically significant.

## Results

### Types of *S. aureus* carriers

It was found that only 6.2% of the sampled persons were not carriers of *S. aureus*, 18.3% were persistent carriers in both anatomical sites, 19.3% were exclusive persistent carriers in the pharynx and no exclusive persistent carriers were found in the nose. Furthermore, 15.3% were intermittent carriers in both the pharynx and nose), only 5.2% were found to be persistent carriers in the nose and intermittent carriers in the pharynx and another 15.3% of the students were persistent carriers in the pharynx and intermittent carriers in the nose, while 20.4% of the persons are exclusive intermittent carriers in the pharynx; finally, no exclusive intermittent carriers in the nose were found (Table 1). No significant differences were found between *S. aureus* carriers with respect to age and gender (*P*>0.05).

### Distribution of staphylococcal strains present in the nose and pharynx

**Fig. 1.**
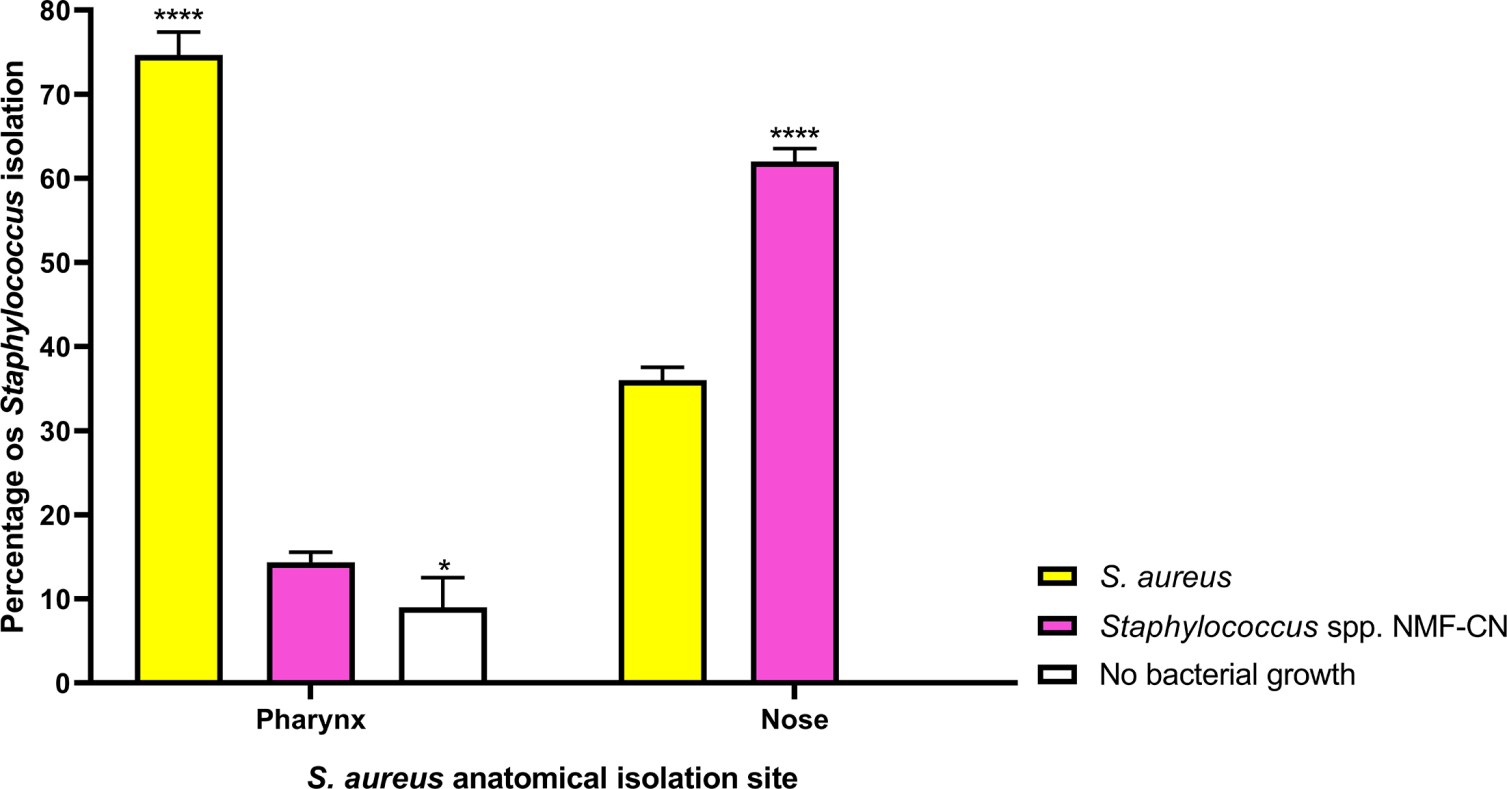
Distribution of *S. aureus* and *Staphylococcus* spp. strains isolated from the pharynx and nose. The bars are the average percentage of *S. aureus* and *Staphylococcus* spp. strains isolated in the pharynx and nose from the three samplings performed; the error bars represent the sem (*P<0.05, ****P<0.0001). NMF-CN, mannitol-coagulase-negative non-fermenting staphylococci.

74.6% of *S. aureus* strains were isolated from the pharynx and the remaining 36% were isolated from the nose (*P*<0.0001), while only 14.3% of *Staphylococcus* spp. strains were found in the pharynx and 62% in the nose (*P*<0.0001). It is important to mention that in all cultures of nasal exudates, there was always growth of some *Staphylococcus* spp. strain, while in the pharynx, 9% (±6.0) of the cultures showed no growth of *Staphylococcus* spp. strains (*P*=0.029) (Fig. 1). Twenty-two MRSA strains (7.02%) were isolated, of which 12 were isolated from the pharynx (3.83%) and 10 from the nose (3.19%).

### Alpha-diversity analysis

Table 2 presents the OTUs, coverage and alpha-diversity analysis of inverse Simpson’s, Shannon’s and Chao’s indices of the six *S. aureus* carrier groups. The metagenomic coverage was very similar for the six *S. aureus* carrier groups; on the other hand, Table 2 also shows that the pharyngeal microbiomes were shown to have a better uniformity of organisms, but lower diversity compared to the nasal bacterial communities.

### Analysis of bacterial relative abundance

#### Metataxonomic comparison of the phylum-level microbiome of *S. aureus* carriers

**Fig. 2.**
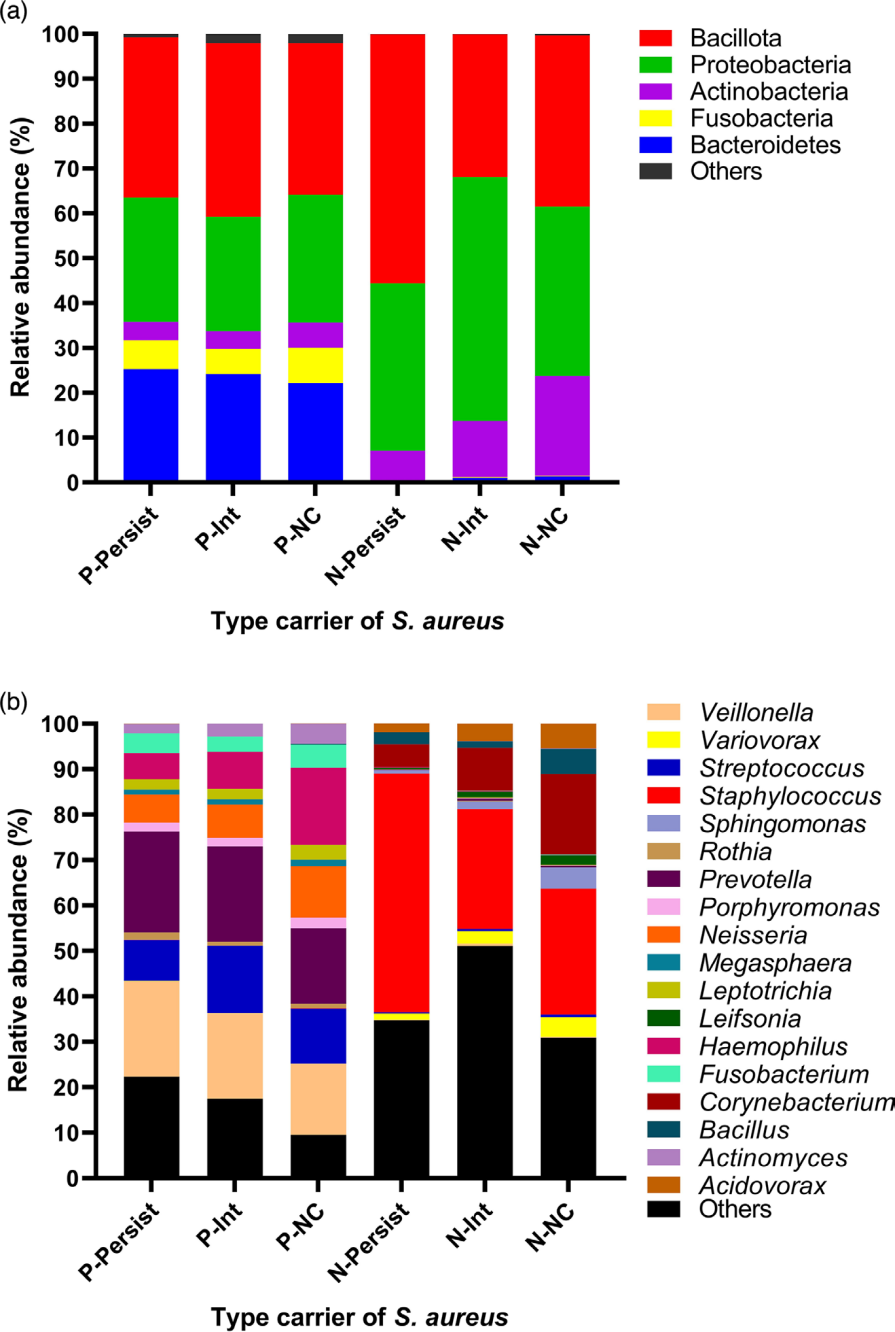
Percentages of relative bacterial abundances of the microbiomes of *S. aureus* carriers and non-carriers in the pharynx and nose. (a) Phylum level. (b) Genus level. Bars represent the average relative abundance. P-Persist, persistent carrier in the pharynx; P-Int, intermittent carrier in the pharynx; P-NC, non-carrier in the pharynx; N-Persist, persistent carrier in the nose; N-Int, intermittent carrier in the nose; N-NC, non-carrier in the nose.

Metataxonomic analysis revealed 19 bacterial phyla in the pharynx and 18 in the nose; however, there were no statistically significant differences when comparing the phyla with relative abundances greater than 1% found in the microbiomes of P-Persist, P-Int and P-NC carriers, not even when comparing the microbiomes of the N-Persist, N-Int and N-NC (Fig. 2a, Table 3). Five predominant phyla were found, the phyla *Bacillota*, *Proteobacteria*, *Bacteroidetes*, *Fusobacteria* and *Actinobacteria* (Fig. 2a). The phyla *Bacteroidetes* and *Fusobacteria* are more abundant in the pharynx than in the nose SP (*P*<0.0001), while the phylum *Actinobacteria* is more abundant in the nasal microbiome compared to the pharyngeal microbiome (*P*<0.043). It is important to note that the phylum *Bacillota* (which includes *Staphylococcus*) was found in higher proportion in the N-Persist (55.55%) (*P*>0.05), compared to 31.84% in the N-Int and 38.22% in the N-NC. A similar pattern occurs with the phylum *Proteobacteria* (higher abundance in the N-Int, compared to the P-Persist and N-NC). When comparing specifically the N-Persist versus N-NC microbiomes, only *Bacteroidetes* are in greater abundance in the N-NC (*P*<0.001).

**Table 3.** Relative abundances of the main bacterial phyla, orders, families and genera that are statistically significant in the six types of *S. aureus* carriers in the pharynx and nose

Taxon	*P-*value	P-Persist	P-Int	P-NC	N-Persist	N-Int	N-NC
	**Percentages of relative abundance**						
**Phylum**							
*Actinobacteria*	4.3e−2	4.08	3.95	5.61	6.76	12.54	22.25
*Bacteroidetes*	1.1e−6	25.23	24.19	22.15	0.24	1.05	1.35
*Bacillota*	n.s	35.73	38.68	33.84	55.55	31.84	38.22
*Fusobacteria*	2e−4	6.48	5.59	7.88	0.03	0.14	0.12
*Proteobacteria*	n.s	27.68	25.50	28.47	37.34	54.29	37.73
**Order**							
*Actinomycetales*	3.4e−2	3.65	3.51	5.09	6.73	12.48	22.16
*Bacillales*	2e−5	1.40	1.42	1.19	54.44	27.18	32.52
*Bacteroidales*	1.6e−6	24.90	23.76	20.80	0.09	0.81	0.50
*Burkholderiales*	5e−4	0.14	0.03	0.10	3.35	6.65	10.37
*Clostridiales*	2.9e−2	3.24	2.94	3.53	0.62	1.39	1.27
*Fusobacteriales*	2e−4	6.49	5.61	7.89	0.03	0.14	0.12
*Lactobacillales*	5e−3	9.26	15.03	12.33	0.37	2.70	4.17
*Neisseriales*	1.9e−2	6.06	6.87	10.60	0.06	0.20	0.29
*Pasteurellales*	1.8e−2	5.71	7.85	16.46	0.09	0.15	0.08
*Selenomonadales*	8.9e−6	21.68	18.95	16.64	0.11	0.56	0.28
*Sphingomonadales*	8.6e−6	0.03	0.02	0.03	0.87	1.73	4.94
**Family**							
*Actinomycetaceae*	5.6e−5	1.97	2.70	4.11	0.04	0.10	0.11
*Comamonadaceae*	8e−4	0.12	0.02	0.07	3.27	6.62	9.76
*Corynebacteriaceae*	1.9e−2	0.01	0.01	0.01	5.03	9.32	17.57
*Fusobacteriaceae*	7e−3	4.18	3.24	4.85	0.02	0.06	0.08
*Lachnospiraceae*	1.5e−4	1.88	2.02	2.49	0.04	0.22	0.20
*Leptotrichiaceae*	6.5e−6	2.34	2.45	3.09	0.02	0.09	0.05
*Moraxellaceae*	6.1e−3	2.89	0.03	0.41	2.79	4.52	16.18
*Neisseriaceae*	1.8e−2	6.08	7.05	10.70	0.06	0.21	0.30
*Pasteurellaceae*	1.7e−2	5.74	8.01	16.52	0.10	0.16	0.09
*Porphyromonadaceae*	3.7e−2	1.95	1.88	2.23	0.01	0.10	0.10
*Prevotellaceae*	1.3e−5	22.86	22.12	18.30	0.07	0.71	0.39
*Sphingomonadaceae*	9.5e−6	0.03	0.03	0.04	0.87	1.76	5.00
*Staphylococcaceae*	5e−4	0.09	0.04	0.10	52.31	26.08	27.48
*Streptococcaceae*	1e−3	8.63	14.29	11.42	0.29	0.48	0.59
*Veillonellaceae*	9.2e−6	21.77	19.39	16.71	0.12	0.56	0.29
**Genus**							
*Acidovorax*	8e−4	0.067	0.01	0.048	1.87	3.84	5.43
*Actinomyces*	4.4e−5	2.02	2.82	4.36	0.036	0.091	0.113
*Bacillus*	1e−3	0.018	0.01	0.161	2.62	1.36	5.53
*Corynebacterium*	1.9e−2	0.008	0.008	0.005	5.06	9.45	17.74
*Fusobacterium*	7e−3	4.36	3.38	5.09	0.019	0.063	0.079
*Haemophilus*	1.4e−2	5.72	8.11	16.99	0.093	0.157	0.09
*Leifsonia*	1e−3	0.01	0.006	0.019	0.35	1.21	2.05
*Leptotrichia*	1.7e−5	2.34	2.33	3.25	0.013	0.079	0.047
*Megasphaera*	8e−3	1.02	1.10	1.48	0.006	0.066	0.002
*Neisseria*	1.6e−2	6.22	7.34	11.28	0.029	0.064	0.076
*Porphyromonas*	4.7e−2	1.94	1.88	2.32	0.008	0.096	0.091
*Prevotella*	1.6e−5	22.19	20.99	16.61	0.07	0.525	0.35
*Rothia*	4.3e−2	1.6	0.84	1	0.015	0.127	0.029
*Sphingomonas*	1.1e−5	0.027	0.004	0.024	0.821	1.67	4.71
*Staphylococcus*	5e−4	0.094	0.046	0.103	52.47	26.36	27.68
*Streptococcus*	8e−4	8.95	14.79	12.06	0.29	0.468	0.583
*Variovorax*	8e−4	0.053	0.004	0.015	1.38	2.78	4.31
*Veillonella*	1e−5	21.03	18.85	15.65	0.075	0.471	0.187

Multiple comparison ANOVA followed by Tukey-Kramer post hoc test was performed.

N-Intintermittent carrier in noseN-NCnon-carrier in noseN-Persist, persistent carrier in nose; n.snot significantP-Int, intermittent carrier in pharynx; P-NC, non-carrier in pharynx; P-Persist, persistent carrier in pharynx

When the phyla of microbiomes with an abundance of less than 1% were analysed, it was found that the phylum *Acidobacteria* showed a higher abundance in N-Persist and N-NC compared to N-Int with a significant difference (*P*<0.001). In addition, the phyla *Planctomyces* and *Chloroflexi* also showed a higher abundance in N-NC than in N-Persist and N-Int (*P*<0.01). Likewise, it was found that P-NC presented a higher abundance of the phyla *Deinococcus-Thermus* and *Tenericutes* compared to P-Persist and P-int (*P*<0.01) (Table 3).

These results show that the main differences in composition of phyla in the microbiome are due to anatomical site rather than carrier status (Fig. 2a). That is, no significant difference is found between the microbiomes at the phyla level of the different types of carriers, whether from the nose or pharynx, but there are differences with respect to the anatomical niche, which can be observed in Table 3, which presents the relative abundance data of the four types of carriers and two types of non-carriers of * S. aureus* in the pharynx and nose, as well as the *P*-values at different taxonomic levels.

#### Metataxonomic comparison of the genus-level microbiome of *S. aureus* carriers

When analysing the pharyngeal microbiome at the genus level, up to 178 genera are found, while the nasal microbiomes showed up to 248 different genera, confirming a greater variety of organisms than shown in the alpha-diversity analysis compared to the pharyngeal microbiome (Table 2). The most abundant genera in the pharynx are *Prevotella*, *Veillonella*, *Streptococcus*, *Haemophilus*, *Neisseria*, *Fusobacterium*, *Porphyromonas*, *Leptotrichia* and *Actinomyces*, while *Staphylococcus*, *Corynebacterium*, *Acidovorax*, *Bacillus*, *Sphingomonas* and *Variovorax* are the genera with the highest abundance in the nose (Fig. 2b, Table 3). There are no significant differences with respect to the microbiome genera with abundances greater than 1% when comparing the P-Persist, P-Int and N-NC microbiomes, nor between the N-Persist, N-Int and N-NC microbiomes (Fig. 2b, Table 3).

It is important to note that the genus *Staphylococcus* does not show statistical difference in any of the microbiomes of the pharyngeal or nasal carrier groups (Fig. 2b, Table 3), and the relative abundance is 0.094% in P-Persist, 0.046% in P-Int and 0.103% in P-NC, while there are 52.47% in N-Persist, 26.36% in N-Int and 27.68 % in N-NC, being a scarce genus in the pharyngeal microbiome despite the presence of persistent and intermittent carriers of *S. aureus*.

However, when comparing the low and very low abundance genera, if significant changes are found in the composition of the microbiome at the genus level, 35 genera were found present in the P-NC, which are absent in the pharyngeal carriers of *S. aureus* (P-Persist and P-Int) (*P*<0.05 and <0.001), so at low abundances, there is a difference in the pharyngeal microbiome between * S. aureus* carriers compared to non-carriers (Table 4). Regarding the nasal microbiome, not many differences were found in the low and very low abundance genera between carriers and non-carriers; only the *Pontibacter* and *Rhizobacter* genera are exclusively present in N-NC (*P*<0.05) (Table 5).

**Table 4.** Relative abundances of the main bacterial phyla, orders, families and genera that are statistically significant in the *S. aureus* carriers and non-carriers in the pharynx

Taxon	*P-*value	P-Persist	P-Int	P-NC
	Percentage of relative abundance			
**Phylum**				
*Actinobacteria*	n.s	4.08	3.95	5.61
*Bacteroidetes*	n.s	25.23	24.19	22.15
*Bacillota*	n.s	35.73	38.68	33.84
*Fusobacteria*	n.s	6.48	5.59	7.88
*Proteobacteria*	n.s	27.68	25.50	28.47
*Deinococcus-Thermus*	8.6e−3	0	2.7e−4	1.9e−3
*Tenericutes*	0.04	9.2e−4	2.7e−3	0.024
**Order**				
*Actinomycetales*	n.s	3.65	3.51	5.09
*Bacillales*	n.s	1.40	1.42	1.19
*Bacteroidales*	n.s	24.90	23.76	20.80
*Burkholderiales*	n.s	0.14	0.03	0.10
*Clostridiales*	n.s	3.24	2.94	3.53
*Fusobacteriales*	n.s	6.49	5.61	7.89
*Lactobacillales*	n.s	9.26	15.03	12.33
*Neisseriales*	n.s	6.06	6.87	10.60
*Pasteurellales*	n.s	5.71	7.85	16.46
*Selenomonadales*	n.s	21.68	18.95	16.64
*Sphingomonadales*	n.s	0.03	0.02	0.03
*Vibrionales*	1.5e−3	2.6e−4	0	2.6e−3
*Deinococcales*	8.7e−3	0	2.7e−4	1.9e−3
*Hydrogenophilales*	0.016	4.8e−4	0	4.4e−3
*Mycoplasmatales*	0.038	6.3e−4	2.7e−3	0.024
*Alphaproteobacteria*	0.040	0	0	1.2e−3
*Rickettsiales*	0.040	0	0	6.6e−5
*Chromatiales*	0.040	0	0	6.6e−5
*Oceanospirillales*	0.040	0	0	2.5e−3
**Family**				
*Actinomycetaceae*	n.s	1.97	2.70	4.11
*Comamonadaceae*	n.s	0.12	0.02	0.07
*Corynebacteriaceae*	n.s	0.01	0.01	0.01
*Fusobacteriaceae*	n.s	4.18	3.24	4.85
*Lachnospiraceae*	n.s	1.88	2.02	2.49
*Leptotrichiaceae*	n.s	2.34	2.45	3.09
*Moraxellaceae*	n.s	2.89	0.03	0.41
*Neisseriaceae*	n.s	6.08	7.05	10.70
*Pasteurellaceae*	n.s	5.74	8.01	16.52
*Porphyromonadaceae*	n.s	1.95	1.88	2.23
*Prevotellaceae*	n.s	22.86	22.12	18.30
*Sphingomonadaceae*	n.s	0.03	0.03	0.04
*Staphylococcaceae*	n.s	0.09	0.04	0.10
*Streptococcaceae*	n.s	8.63	14.29	11.42
*Veillonellaceae*	n.s	21.77	19.39	16.71
*Deinococcaceae*	4.7e−4	0	0	1.9e−3
*Methylobacteriaceae*	1.1e−3	4.8e−4	8.2e−4	5.2e−3
*Vibrionaceae*	1.5e−3	2.6e−4	0	2.6e−3
*Oxalobacteraceae*	6.7e−3	1.4e−3	8.2e−4	0.011
*Nocardioidaceae*	0.011	6.4e−4	0	2.6e−3
*Hydrogenophilaceae*	0.016	4.8e−4	0	4.5e−3
*Mycoplasmataceae*	0.038	6.4e−4	2.8e−3	0.024
*Alphaproteobacteria_family_incertae_sedis*	0.04	0	0	1.2e−3
*Rhizobiaceae*	0.04	0	0	6.7e−4
*Bacillaceae*	0.04	0	0	6.7e−4
*Granulosicoccaceae*	0.04	0	0	6.7e−4
*Pseudonocardiaceae*	0.04	0	0	6.7e−4
*Syntrophaceae*	0.04	0	0	6.7e−4
*Iamiaceae*	0.04	0	0	6.7e−4
*Aurantimonadaceae*	0.04	0	0	6.7e−4
*Hyphomicrobiaceae*	0.04	0	0	1.2e−3
*Rickettsiaceae*	0.04	0	0	6.7e−4
*Halomonadaceae*	0.04	0	0	2.5e−3
*Brucellaceae*	0.04	0	0	6.7e−4
**Genus**				
*Acidovorax*	8e−4	0.067	0.01	0.048
*Actinomyces*	4.4e−5	2.02	2.82	4.36
*Bacillus*	4.6e−3	0.018	0.01	0.161
*Corynebacterium*	1.9e−2	0.008	0.008	0.005
*Fusobacterium*	7e−3	4.36	3.38	5.09
*Haemophilus*	1.4e−2	5.72	8.11	16.99
*Leifsonia*	1e−3	0.01	0.006	0.019
*Leptotrichia*	1.7e−5	2.34	2.33	3.25
*Megasphaera*	8e−3	1.02	1.10	1.48
*Neisseria*	1.6e−2	6.22	7.34	11.28
*Porphyromonas*	4.7e−2	1.94	1.88	2.32
*Prevotella*	1.6e−5	22.19	20.99	16.61
*Rothia*	4.3e−2	1.6	0.84	1
*Sphingomonas*	1.1e−5	0.027	0.004	0.024
*Staphylococcus*	5e−4	0.094	0.046	0.103
*Streptococcus*	8e−4	8.95	14.79	12.06
*Variovorax*	8e−4	0.053	0.004	0.015
*Veillonella*	1e−5	21.03	18.85	15.65
*Roseomonas*	1.2e−4	0	0	1.4e−3
*Trichococcus*	4e−4	0	0	2e−3
*Deinococcus*	4e−4	0	0	2e−3
*Escherichia_Shigella*	4e−4	0	0	2e−3
*Arthrobacter*	4.5e−4	0	0	2e−3
*Massilia*	9.8e−4	2.7e−4	5.9e−4	1.1e−2
*Methylobacterium*	1.1e−3	5e−4	8.7e−4	5.5e−3
*Vibrio*	1.4e−3	2.7e−4	0	2.8e−3
*Wautersiella*	6.8e−3	8e−4	1e−3	1.5e−2
*Phenylobacterium*	9.6e−3	1e−3	0	4.1e−3
*Cloacibacterium*	0.014	0	5e−4	2e−3
*Hydrogenophilus*	0.015	5e−4	0	4.7e−3
*Nocardioides*	0.024	3e−4	0	2e−3
*Ureaplasma*	0.040	0	0	2.3e−2
*Nevskia*	0.040	0	0	1.3e−3
*Rhizobium*	0.040	0	0	7.3e−4
*Chitinibacter*	0.040	0	0	7.1e−4
*Iamia*	0.040	0	0	7.1e−4
*Smithella*	0.040	0	0	7.1e−4
*Actinomycetospora*	0.040	0	0	7.3e−4
*Hydrogenophaga*	0.040	0	0	1.3e−3
*Fervidicella*	0.040	0	0	7.3e−4
*Rhodopila*	0.040	0	0	7.1e−4
*Halomonas*	0.040	0	0	2.6e−3
*Geminicoccus*	0.040	0	0	1.3e−3
*Ignatzschineria*	0.040	0	0	7.3e−4
*Faecalibacterium*	0.040	0	0	7.1e−4
*Paraprevotella*	0.040	0	0	7.3e−4
*Azospirillum*	0.040	0	0	7.1e−4
*Burkholderia*	0.040	0	0	7.3e−4
*Aurantimonas*	0.040	0	0	7.1e−4
*Granulosicoccus*	0.040	0	0	7.1e−4
*Dyadobacter*	0.040	0	0	1.3e−3
*Flectobacillus*	0.040	0	0	7.1e−4
*Comamonas*	0.040	0	0	7.3e−4
*Dyella*	0.040	0	0	1.3e−3
*Vampirovibrio*	0.040	0	0	7.1e−4
*Azonexus*	0.040	0	0	7.1e−4
*Clostridium*	0.040	0	0	1.3e−3
*Devosia*	0.040	0	0	1.3e−3
*Achromobacter*	0.040	0	0	7.1e−4
*Pelomonas*	0.040	0	0	2.6e−3
*Moraxella*	0.040	0	2.2e−4	1.4e−1
*Roseomonas*	0.040	0	0	1.4e−3

Multiple comparison ANOVA followed by Tukey-Kramer post-hoc test was performed.

n.snot significantP-Int, intermittent carrier in pharynx; P-NCnoncarrier in pharynxP-Persist, persistent carrier in pharynx

**Table 5.** Relative abundances of the main bacterial phyla, orders, families and genera that are statistically significant in the *S. aureus* carriers and non-carriers in the nose

Taxon	*P-*value	N-Persist	N-Int	N-NC
	Percentage of relative abundance			
**Phylum**				
*Actinobacteria*	n.s	6.76	12.54	22.25
*Bacteroidetes*	n.s	0.24	1.05	1.35
*Bacillota*	n.s	55.55	31.84	38.22
*Fusobacteria*	n.s	0.03	0.14	0.12
*Proteobacteria*	n.s	37.34	54.29	37.73
*Acidobacteria*	7.1e−4	0.021	1.8e−2	0.085
*Planctomyces*	3.2e−3	2.6e−3	7.2e−3	0.045
*Chloroflexi*	4.9e−3	6.3e−3	9.1e−3	0.048
**Order**				
*Actinomycetales*	n.s	6.73	12.48	22.16
*Bacillales*	n.s	54.44	27.18	32.52
*Bacteroidales*	n.s	0.09	0.81	0.50
*Burkholderiales*	n.s	3.35	6.65	10.37
*Clostridiales*	n.s	0.62	1.39	1.27
*Fusobacteriales*	n.s	0.03	0.14	0.12
*Lactobacillales*	n.s	0.37	2.70	4.17
*Neisseriales*	n.s	0.06	0.20	0.29
*Pasteurellales*	n.s	0.09	0.15	0.08
*Selenomonadales*	n.s	0.11	0.56	0.28
*Sphingomonadales*	0.025	0.87	1.73	4.94
*Solirubrobacterales*	1e−5	1.8e−3	3.2e−3	0.039
*Planctomycetales*	2.8e−3	2.6e−3	6.2e−3	0.045
*Vibrionales*	4.2e−3	4.2e−3	0.016	0.049
*Caulobacterales*	5.9e−3	0.093	0.136	0.706
*Verrucomicrobiales*	7.1e−3	3.7e−3	4e−3	0.024
*Flavobacteriales*	0.01	0.104	0.166	0.603
*Sphingobacteriales*	0.015	0.041	0.06	0.204
*Rhizobiales*	0.025	0.293	0.55	1.49
*Bacteroidetes_inc*	0.039	2.4e−3	1.8e−3	0.03
*Chromatiales*	0.045	2.6e−3	2.9e−3	0.019
**Family**				
*Actinomycetaceae*	n.s	0.04	0.10	0.11
*Comamonadaceae*	n.s	3.27	6.62	9.76
*Corynebacteriaceae*	n.s	5.03	9.32	17.57
*Fusobacteriaceae*	n.s	0.02	0.06	0.08
*Lachnospiraceae*	n.s	0.04	0.22	0.20
*Leptotrichiaceae*	n.s	0.02	0.09	0.05
*Moraxellaceae*	0.014	2.79	4.52	16.18
*Neisseriaceae*	n.s	0.06	0.21	0.30
*Pasteurellaceae*	n.s	0.10	0.16	0.09
*Porphyromonadaceae*	n.s	0.01	0.10	0.10
*Prevotellaceae*	n.s	0.07	0.71	0.39
*Sphingomonadaceae*	0.026	0.87	1.76	5.00
*Staphylococcaceae*	n.s	52.31	26.08	27.48
*Streptococcaceae*	n.s	0.29	0.48	0.59
*Veillonellaceae*	n.s	0.12	0.56	0.29
*Hyphomicrobiaceae*	8.8e−5	0.012	0.011	0.076
*Mycobacteriaceae*	9e−4	1.4e−3	1.8e−3	0.019
*Planctomycetaceae*	3e−3	2.7e−3	6.4e−3	0.046
*Planococcaceae*	3.3e−3	4.8e−3	3.5e−3	0.021
*Vibrionaceae*	4.3e−2	4.3e−3	0.017	0.050
*Burkholderiaceae*	4.8e−3	0.038	0.054	0.267
*Caulobacteraceae*	5.9e−3	0.095	0.139	0.723
*Nocardioidaceae*	6.7e−3	0.022	0.023	0.091
*Verrucomicrobiaceae*	6.7e−3	3.7e−3	4e−3	0.025
*Methylobacteriaceae*	6.9e−3	0.020	0.040	0.190
*Methylocystaceae*	7e−3	3.4e−3	0	0.032
*Bacillales*	7.1e−3	1.9e−3	2.1e−3	0.012
*Flavobacteriaceae*	9e−3	0.104	0.165	0.612
*Alcaligenaceae*	0.012	1.7e−3	5.4e−3	0.028
*Oxalobacteraceae*	0.012	0.081	0.135	0.534
*Alteromonadaceae*	0.014	1.8e−3	2.7e−4	0.010
*Kineosporiaceae*	0.018	3.4e−4	0	8.4e−3
*Geodermatophilaceae*	0.021	8.1e−3	0.013	0.046
*Cytophagaceae*	0.023	0.014	6.6e−3	0.069
*Enterococcaceae*	0.027	0.020	0.018	0.084
*Erythrobacteraceae*	0.030	0	3.9e−3	0.018
*Peptostreptococcaceae*	0.033	8e−3	0.024	0.060
*Chitinophagaceae*	0.038	0.017	0.042	0.117
*Bacteroidetes*	0.039	2.5e−3	1.8e−3	0.03
**Genus**				
*Acidovorax*	8e−4	1.87	3.84	5.43
*Actinomyces*	4.4e−5	0.036	0.091	0.113
*Bacillus*	1e−3	2.62	1.36	5.53
*Corynebacterium*	1.9e−2	5.06	9.45	17.74
*Fusobacterium*	7e−3	0.019	0.063	0.079
*Haemophilus*	1.4e−2	0.093	0.157	0.09
*Leifsonia*	1e−3	0.35	1.21	2.05
*Leptotrichia*	1.7e−5	0.013	0.079	0.047
*Megasphaera*	8e−3	0.006	0.066	0.002
*Neisseria*	1.6e−2	0.029	0.064	0.076
*Porphyromonas*	4.7e−2	0.008	0.096	0.091
*Prevotella*	1.6e−5	0.07	0.525	0.35
*Rothia*	4.3e−2	0.015	0.127	0.029
*Sphingomonas*	0.028	0.821	1.67	4.71
*Staphylococcus*	5e−4	52.47	26.36	27.68
*Streptococcus*	8e−4	0.29	0.468	0.583
*Variovorax*	8e−4	1.38	2.78	4.31
*Veillonella*	1e−5	0.075	0.471	0.187
*Devosia*	1.6e−4	8.8e−3	2.1e−3	6e−2
*Marmoricola*	6e−4	5.3e−3	3.1e−3	3e−2
*Mycobacterium*	9.2e−4	1.4e−3	1.9e−3	1.9e−2
*Singulisphaera*	1e−3	1.4e−3	1.4e−3	2.4e−2
*Alloiococcus*	1.3e−3	4.6e−3	1.4e−3	5.4e−2
*Vibrio*	3.2e−3	2.8e−3	1.5e−2	5e−2
*Flavobacterium*	3.4e−3	3.6e−3	7.3e−3	5.9e−2
*Cupriavidus*	4.3e−3	0.033	4.3e−2	2.6e−1
*Wautersiella*	4.5e−3	0.076	0.10	4.6e−1
*Achromobacter*	5.6e−3	1.3e−3	4.7e−3	2.3e−2
*Methylobacterium*	5.6e−3	0.019	3.7e−2	1.9e−1
*Brevundimonas*	5.7e−3	0.07	9.1e−2	5.1e−1
*Pelomonas*	6.2e−3	2.9e−3	8e−3	4e−2
*Exiguobacterium*	8e−3	1.9e−3	2.1e−3	1.2e−2
*Coprococcus*	9.8e−3	0	1.1e−3	1e−2
*Phenylobacterium*	9.9e−3	0.019	3.8e−2	1.7e−1
*Lysobacter*	0.011	5e−4	1e−3	1e−2
*Alishewanella*	0.012	1.8e−3	0	1e−2
*Porphyrobacter*	0.014	0	1e−3	9.7e−3
*Psychrobacter*	0.015	0.014	2.2e−1	8e−1
*Massilia*	0.015	0.064	0.1	4e−1
*Blastococcus*	0.017	7.5e−3	1.2e−2	4.3e−2
*Sediminibacterium*	0.017	7.5e−3	2.5e−2	8.7e−2
*Undibacterium*	0.019	8.6e−3	2.2e−2	9.8e−2
*Enterococcus*	0.019	1.8e−2	1.9e−2	8.5e−2
*Acinetobacter*	0.023	2.62	4.19	12.46
*Novosphingobium*	0.024	5e−2	7.6e−2	0.318
*Rhizobacter*	0.032	0	0	5.9e−3
*Ohtaekwangia*	0.039	2.5e−3	0.019	0.031
*Pontibacter*	0.045	0	0	7.3e−3
*Nocardioides*	0.047	0.014	0.015	0.048
*Jeotgalicoccus*	0.048	0.011	0.011	0.04

Multiple comparison ANOVA followed by Tukey-Kramer post-hoc test was performed.

N-Int, intermittent carrier in nose; N-NCnoncarrier in noseN-Persist, persistent carrier in nosen.snot significant

Interestingly, although more *S. aureus* carriers were found in the pharynx than in the nose by traditional microbiological analysis, metataxonomic analysis revealed a higher presence of the genus *Staphylococcus* in the nose than in the pharynx. There are no significant differences between the P-Persist, P-Int and N-NC microbiomes, nor between the N-Persist, N-Int and N-NC microbiomes (Fig. 2a, b, Table 3). In this regard, microbiological analysis showed that, although fewer *S. aureus* strains were isolated from the nose than from the pharynx, other species of the genus *Staphylococcus* colonize the human nose (Fig. 2b).

### Principal component analysis of the pharyngeal and nasal microbiomes of *S. aureus* carriers

**Fig. 3.**
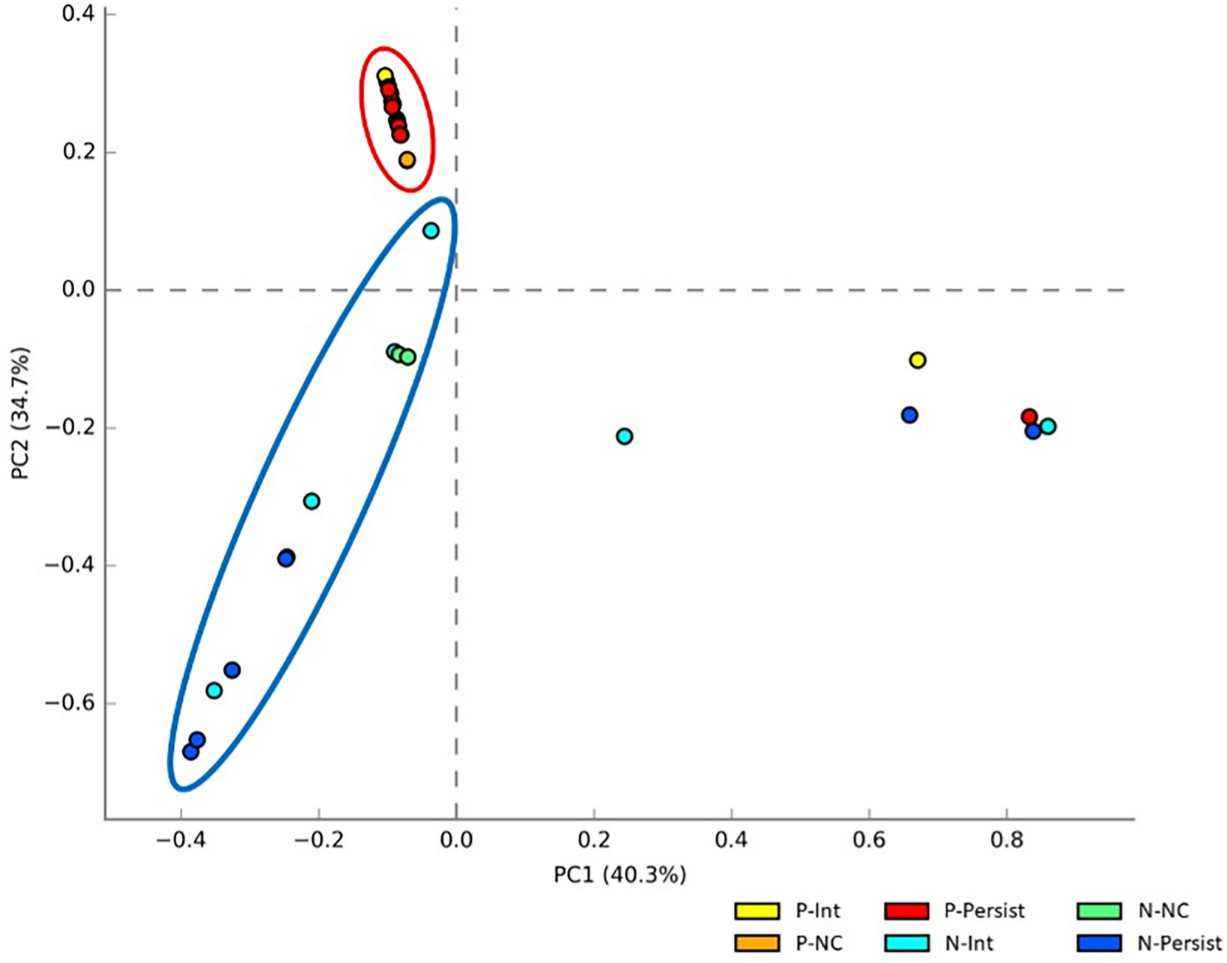
PCA plot of SP of *S. aureus* carrier types in the pharynx and nose at bacterial genus level. P-Persist, persistent carrier in the pharynx; P-Int, intermittent carrier in the pharynx; P-NC, non-carrier in the pharynx; N-Persist, persistent carrier in the nose; N-Int, intermittent carrier in the nose; N-NC, non-carrier in the nose.

The differences and similarities in the bacterial composition of the pharyngeal and nasal microbiome of *S. aureus* carriers and non-carriers can be seen in Fig. 3, which presents a principal component analysis (PCA) plot at the genus level, where 19 of the 21 pharyngeal SPs are clustered in the upper left quadrant of PC2, with two atypical SPs (one from the P-Int group and one from P-Persist), showing relative closeness to each other. PCA plots of the order and family levels were made (Figs S1 and S2), where similar results to those shown in the PCA of genus are observed. This indicates that the composition of the microbiome in the pharyngeal samples of *S. aureus* carriers and non-carriers is very similar (Table 4). Besides, the 15 nasal SPs show greater dispersion than the pharyngeal SP (Fig. 3), with 4 atypical nasal pools (lower right quadrant of PC1), reaffirming the differences noted in the composition of the nasal microbiome in the relative abundance analysis at the phylum and genus levels (Fig. 2, Table 3). Although almost all pharyngeal and nasal SP clustered in the same sectors, five SPs, two pharyngeal and three nasals, show atypical behaviour, with little variability between them. It is also important to note the clear division of the composition of the pharyngeal microbiome and the nasal microbiome as seen in Figs2  3 and Tables3^5^.

### Prediction of functional analysis of pharyngeal and nasal microbiome of *S. aureus* carriers and non-carriers

**Fig. 4.**
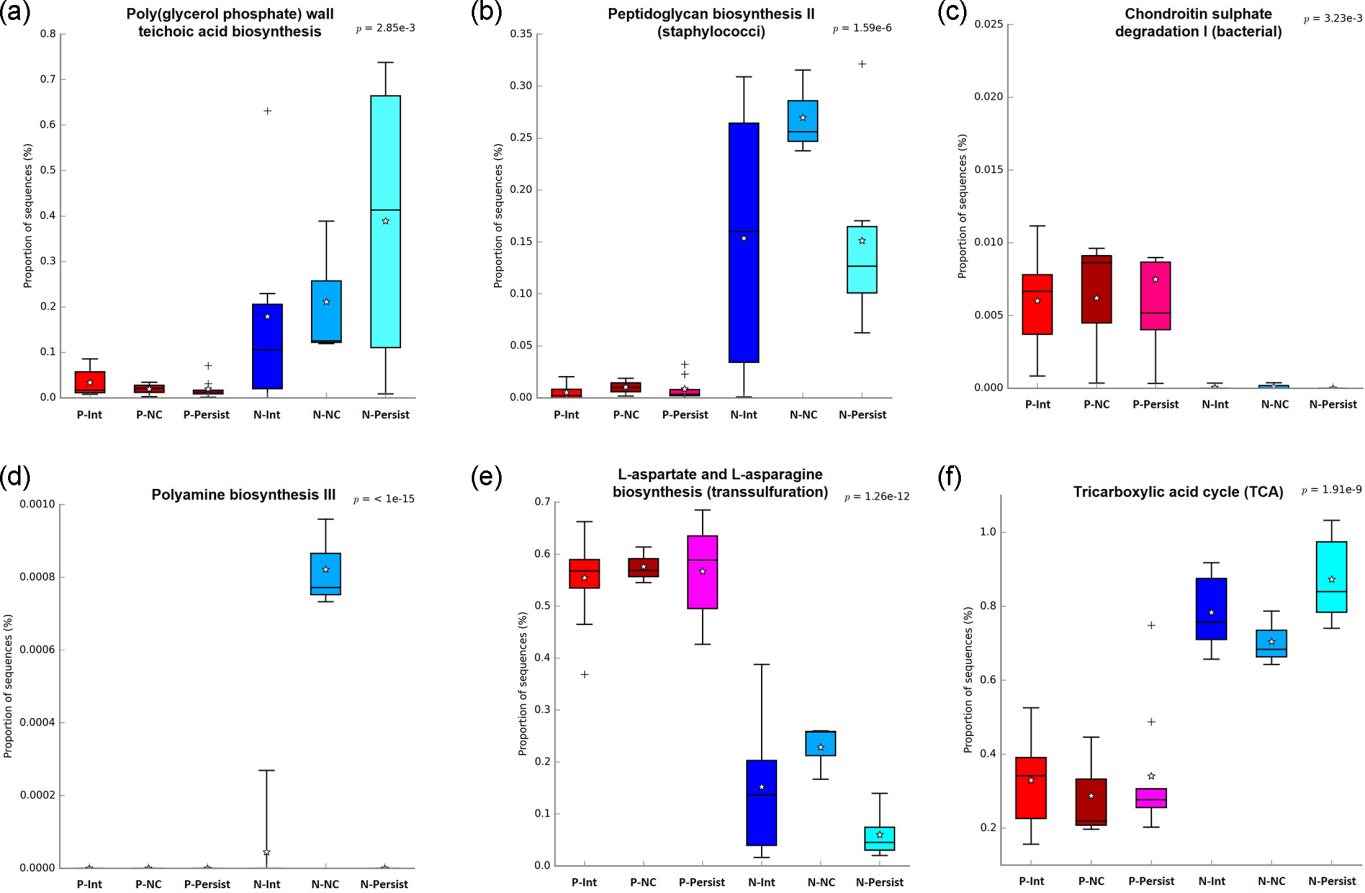
Box plot of the phylogenetic investigation of bacterial communities (PICRUSt2) of KEGG metabolic pathways. (a) Biosynthesis of teichoic acids from poly(glycerol phosphate), (b) biosynthesis of peptidoglycan II (staphylococci), (c) degradation of chondroitin sulphate I (bacterial), (d) polyamine biosynthesis III, (e) l-aspartate and l-asparagine biosynthesis (transsulfuration) and (f) tricarboxylic acid (TCA) cycle. P-Persist, persistent carrier in the pharynx; P-Int, intermittent carrier in the pharynx; P-NC, non-carrier in the pharynx; N-Persist, persistent carrier in the nose; N-Int, intermittent carrier in the nose; N-NC, non-carrier in the nose.

The prediction of the functional analysis of metabolic pathways of the pharyngeal and nasal microbiomes of *S. aureus* carriers (P-Persist, P-Int, P-NC, N-Persist, N-Int and N-NC) was performed by phylogenetic investigation of communities through reconstruction of unobserved states (PICRUSt2) using the KEGG database. A total of 398 metabolic pathways were identified in the analysis of all types of *S. aureus* carrier types, of which 58 showed statistical difference (Table S2). Fig. 4 shows three pathways that have significant difference between the pharyngeal and nasal microbiomes associated with *Staphylococcus*.

The nasal microbiome has a higher abundance of poly(glycerol phosphate) teichoic acid biosynthesis (Fig. 4a) and peptidoglycan II biosynthesis pathways (staphylococci) (Fig. 4b), and the pharyngeal microbiome has the chondroitin sulphate I degradation pathway (bacterial) and not in the nasal microbiome (Fig. 4c).

Furthermore, three of the pathways with the most significant *P*-values were also analysed: (1) polyamine biosynthesis, (2) l-aspartate and l-asparagine biosynthesis and (3) tricarboxylic acid (TCA) cycle. Polyamines are products of arginine metabolism that present at least two amino groups. This pathway was found in greater proportion in N-NC compared to the groups of nasal carriers (N-Persist and N-Int) and absent in the three groups of pharyngeal carriers (*P*<0.0001) (Fig. 4d). In the case of the biosynthesis of the aas l-aspartate and l-asparagine (Fig. 4e), a greater presence was observed in the three microbiomes of the pharyngeal carriers compared to the nasal carriers (*P*<0.0001). Regarding the TCA cycle pathway, it is found with greater presence in the three microbiomes of nasal carriers compared to the pharyngeal carriers (*P*<0.0001) (Fig. 4f).

## Discussion

Colonization and persistence of *S. aureus* in the pharynx and nose is a process that involves a wide diversity of factors, including pathogen virulence factors, host genetic factors and interactions between *S. aureus* and the host microbiome [46,68]. Although the nose has been reported to be the primary site of *S. aureus* colonization in humans [69,70], there are multiple reports that also point to the pharynx as an important site of colonization, with variable rates of carriers in the pharynx, ranging from 4 to 64%. Some research reports a higher number of carriers in the pharynx than in the nose when samples are collected on the same day [37,38, 41, 46].

In this study, a high percentage of *S. aureus* carriers was found in both the pharynx and nose, as only 6.2% of the people analysed were not carriers of this bacterium. This high percentage of colonization may be because the sampling was performed once a month and both anatomical niches were considered (Fig. 1). It should be noted that all sampled individuals were apparently healthy. These results are similar to those reported by Hanson *et al*. [35], who found a substantially higher percentage of oropharyngeal compared to nasal *S. aureus* carriers (86.1% versus 58.2%). Furthermore, there is ample evidence that parallel *S. aureus* sampling is important, with more pharyngeal than nasal colonization reported [36,40,42]. In this study, no significant differences were found in terms of age or gender with respect to the percentage of carriers; however, there are studies that indicate that there is a difference with respect to gender [71,72].

Hamdan-Partida *et al.* [37] conducted a 5-year longitudinal study of the presence of *S. aureus* in the pharynx of 138 healthy adult workers in a cold meat packing plant in Mexico, sampling them once a year. They found that 18.8% of the volunteers were persistent carriers of *S. aureus* for 3 years or more, 66.7% were intermittent carriers and 14.5% were non-carriers, and in terms of persistence, they report that 12 people were carriers for 3 years, 8 for 4 years, 5 for 5 years and 1 for 6 years. This study also demonstrates that *S. aureus* can persist in the pharynx and nose for shorter periods of time and that intermittency and persistence of *S. aureus* may be more common than previously reported, as *S. aureus* can recolonize both the pharynx and nose within a few weeks. Intermittent carriers may also be a risk factor as mentioned by Ritchie *et al.* [73] and Klyutmans *et al.* [74]. The biological difference between persistent and intermittent strains is that persistent strains may always be the same, while intermittent strains may be different strains with possible different biological characteristics, so their behaviour may vary.

Williamson *et al*. [42] conducted a cross-sectional study on 893 school-aged children in New Zealand; they also report higher colonization in the pharynx (41.1%) than in the nose (31.5%), and 61.3% of children with pharyngeal colonization only carried *S. aureus* exclusively in the pharynx.

The mechanisms of colonization and persistence of *S. aureus* have been extensively studied in the nose. However, the clinical relevance of carriers of this bacterium in the pharynx is poorly recognized and, therefore, has not been sufficiently investigated [75]. This lack of sampling of the pharynx has been justified by considering the nose as the main site of colonization of *S. aureus*, from which it can colonize other parts of the body by manual spread [46,76]. However, as mentioned above, there is sufficient evidence to show that *S. aureus* colonizes and persists better in the pharynx than the human nose [36,40,42].

Besides, while the human oral and nasal microbiomes have been extensively studied, there is little research on the pharyngeal microbiome. Bach *et al*. [4] report the first longitudinal metataxonomic study of the pharyngeal microbiome in healthy individuals, sampling once a week for 40 weeks. They found that the most abundant phylum is the *Bacillota* (particularly the genus *Streptococcus*), followed by *Bacteroidetes*, *Proteobacteria* and *Actinobacteria*. These results are similar to those found in the present study for the three types of pharyngeal carriers of *S. aureus* analysed where the phyla *Bacillota*, *Proteobacteria*, *Bacteroidetes* and *Actinobacteria* were found.

Besides, Bach *et al*. [4] found a moderately high variation in the composition of the microbial communities in the participants over time. It would be interesting to see whether changes in the nasal and pharyngeal microbiomes over time influence *S. aureus* carrier status.

It should be noted that as mentioned in the Results section, no statistically significant differences were found between the microbiomes of the three types of *S. aureus* pharynx carriers in phyla, orders, families and genera with relative abundances greater than 1%. Except for the *Deinococcus* phylum with a higher presence in P-NC with respect to *S. aureus* carriers, it is not the first time that the presence of *Deinococcus-Thermus* has been found in biological samples. Rueca *et al*. [77] reported the presence of *Deinococcus-Thermus* in the nasal/oropharyngeal microbiome in patients negative for Severe Acute Respiratory Syndrome Coronavirus 2 infection. Similarly, Hanshew *et al*. [78] also found *Deinococcus-Thermus* with low abundance in benign vocal lesions. Therefore, despite being an extremophile species, it can be found in the human microbiome.

However, when the genera with an abundance of less than 1% were studied, significant differences were found. There are 38 more genera in non-pharyngeal carriers than in carriers (Table 4). This could indicate that there may be some factors in this niche that allow the presence or not of certain bacterial genera and that possibly facilitate the persistence of *S. aureus*, a fact that we will study in the future.

The genus *Staphylococcus* was found in relative abundances of less than 1% in all three types of *S. aureus* carriers in the pharynx (P-Persist, P-Int and P-NC), so it is not clear why higher abundances were not found in persistent carriers compared to non-carriers and intermittent carriers, nor were differences observed in the abundance of the genera *Prevotella*, *Veillonella* and *Streptococcus*. Only the genus *Haemophilus* was found in higher relative abundance in pharyngeal non-carriers compared to carriers.

The pharyngeal microbiome plays an essential role in the lining of the respiratory tract in protecting against airborne pathogens. However, some pathogens such as *S. aureus*, *S. pneumoniae* and *H. influenzae* can easily colonize the pharynx, becoming part of the microbiome without causing infections in the host [2]. Bogaert *et al*. [79] reported that the abundance of pathogenic bacteria resident in the pharyngeal environment varies seasonally, as does the incidence of respiratory tract infections attributed to them. On the other hand, Moon and Lee [80] reported considerable variation in the pharyngeal microbiome between individuals due to the differences in lifestyle, such as diet, oral hygiene, genetic variations and even random colonization events, with the genus *Streptococcus* predominating.

Although the role of *S. aureus* in the pharyngeal microbiome has been little studied given its importance as a commensal, opportunistic or lethal pathogen, the interactions between *S. aureus* and other bacteria of the pharyngeal microbiome have not been studied in as much detail as in the nose. It is known that each microbial community of pathogens has a threshold of abundance, and if this threshold is exceeded, the risk of infection increases. This threshold indicates that homeostasis is tightly regulated by the microbiome [2,81].

It has been reported that the nasal microbiome is mainly composed of the three bacterial phyla: *Actinobacteria*, *Bacillota* and *Proteobacteria* [12]. The main bacterial genera colonizing the nose are *Staphylococcus*, *Cutibacterium* (formerly *Propionibacterium*), *Corynebacterium* and *Moraxella* [82], while the most common species are *S. aureus*, *Staphylococcus epidermidis* and *Cutibacterium acnes* [83]. Besides, the nasal anatomical environment is nutrient poor, acidic and saline, which constitute stress conditions that nasal microbiome communities must counteract in order to survive and persist [84]. Even though the nose readily allows entry of diverse micro-organisms when in contact with ambient air, there is a clear predominance of genera and species that colonize the upper respiratory tract of healthy adults [85].

Reyes *et al*. [86] conducted a study of the bacterial and fungal nasal microbiome in persistent, intermittent and non-carriers among medical students in Colombia. They reported three predominant phyla, *Proteobacteria*, *Bacillota* and *Actinobacteria*, while the five most abundant genera were *Citrobacter*, *Acinetobacter*, *Corynebacterium*, *Paenibacillum* and *Bacillus*. One of the most important differences between this research and that of Reyes *et al*. [86] is that they found a low abundance of the genus *Staphylococcus*, while our data show high abundance of the genus in both types of nasal carriers, even in non-carriers. The authors mention that a more diverse microbiota may favour the persistence of *S. aureus*; however, they also report no significant differences between the three types of nasal carriers of *S. aureus*. Furthermore, Toro-Ascuy *et al*. [87] carried out microbiome profiles of the nose in healthy adults in Chile, reporting the genus *Staphylococcus* as the most prevalent in the nasal microbiome with a mean of 17.23%, so further research on the nasal microbiome is needed, as it is unclear whether the genus *Staphylococcus* occurs in low or high abundance in the human nasal environment.

Although the genus *Staphylococcus* did not reveal any statistical difference in this investigation between the three types of * S. aureus* nasal carriers, it is important to mention that the relative abundance of the phylum *Bacillota* and the order *Bacillales* (to which *S. aureus* belongs) represents 30–50% of the taxa present in the nasal microbiome of the three types of *S. aureus* carriers (N-Persist, N-Int and N-NC), which coincides with the data obtained from 31.84 to 55.55% in N-Persist.

*S. epidermidis* is the most abundant bacterial species in the human nasal microbiome, although it changes over the years [88,89]. It has been reported that it may protect against colonization by bacterial pathogens and viruses, as it can regulate the immune system of the nose and skin [90]. It is particularly well documented that it can limit the growth, virulence factor expression and colonization of *S. aureus* [91]. *S. epidermidis* has even been used intranasally for the prevention and elimination of MRSA strains [92].

The mechanisms by which *S. epidermidis* reduces the colonization of *S. aureus* and other opportunistic pathogens in the nasal cavity have been extensively studied. These mechanisms include quorum-sensing regulatory molecules, enzyme secretion, molecules with antibiofilm activity, bacteriocins and stimulation of the host innate immune response [83,91]. Notably, *S. epidermidis* is not the only species of the genus that has activity against *S. aureus*; *Staphylococcus hominis* secretes antimicrobial peptides and *S. lugdunensis* produces lugdunin, both of which inhibit the growth of *S. aureus* [21,93].

In this regard, the high abundance of the genus *Staphylococcus* in the nasal microbiomes reported in this work may be due to the high prevalence of non-fermenting mannitol-coagulase-negative staphylococci found in the microbiological cultures (Figs1  2b, Table 3).

Similar to the pharyngeal microbiome, no statistical differences were found in bacterial phyla, orders, families and genera with relative abundances greater than 1% in the three *S. aureus* carriers in the nose (N-Persist, N-Int and N-NC), with the exception of the order *Sphingomonadales* (*P*=0.026) and the families *Moraxellaceae* (*P*=0.014) and *Sphingomonadaceae* (*P*=0.026), being more present in the N-NC than in the N-Persist and N-Int.

As mentioned in the Results section, the main differences observed in the microbiomes are found when comparing the bacterial communities between the two anatomical sites investigated (Fig. 2a, b, Table 3). Each ecological niche offers nutritional and environmental conditions that either favour or inhibit the colonization and persistence of specific bacteria, and their composition may be modified by dysbiosis, antibiotic treatment, etc. These results are consistent with other research where the composition of the microbiome has been found to vary by anatomical site [79,94].

Recently, some research has given more attention to organisms of low abundance under the concept of ‘Keystone Species’, corresponding to micro-organisms whose effect on the microbiome is important in comparison to their low abundance; in this sense, a very abundant species would affect the environment only by its high presence, while a key micro-organism could, for example, influence metabolic functions despite being in very low abundance. Therefore, the identification and study of low abundance organisms in a microbial population can be crucial in the study of diseases or conditions associated with pathogens. These organisms could reveal unique signatures in infectious pathologies or pathogen carriage. However, the current understanding of very low abundance micro-organisms is very limited [95], and the importance of these micro-organisms needs to be further investigated.

When analysing the principal component plot of the pharyngeal and nasal *S. aureus* carriers (Fig. 2), the SPs of the pharyngeal carriers (P-Persist, P-Int and P-NC) cluster in one quadrant, indicating less variation in microbial communities regardless of the type of *S. aureus*, while the nasal microbiome is more prone to present microbiome variations; this could be due to the nasal epithelium being in greater contact with the environment compared to the pharyngeal environment [85], showing that the major differences in the bacterial communities of the microbiome depend mainly on the anatomical site analysed and not on the type of *S. aureus* carrier type in the pharynx or nose (Table 3).

On the other hand, the prediction of PICRUSt2 functional analysis using the KEGG database showed that the staphylococcal cell wall teichoic acid (WTA) biosynthesis pathway is more abundant in either *S. aureus* nasal carrier or nasal non-carrier types (due to *S. epidermidis*, as well as other non-fermenting staphylococci that are mannitol-coagulase negative) (compared to the pharyngeal microbiome (*P*<0.001) (Fig. 4a); this is consistent when comparing the relative abundance of the *Staphylococcus* genus in the nose with respect to the pharynx abundances from 25% in the nose, versus abundances of less than 1% in the pharynx (Table 3).

WTA is an important molecule for *S. aureus* and *S. epidermidis*, as it can interact with various ligands on a large number of tissues and circulatory, epithelial and endothelial cells [scavenger receptor-1 (SREC-1), langerin and Macrophage Galactose-type Lectin], as well as on antibodies and complement activators such as mannose-binding lectin protein, covering diverse functions, including antibacterial effects, and forming interactions that facilitate the colonization and persistence in human cells and tissues [96,97], and WTA is even a potential target as a vaccination antigen [98]. The main receptor reported for WTA is the SREC-1 [99]; however, its expression in the pharynx has not yet been reported [46].

In this work, we found a higher abundance of predicted sequences for the peptidoglycan II (staphylococci) biosynthesis pathway in the nasal microbiome (N-Persist, N-Int and N-NC) compared to the pharyngeal microbiome (P-Persist, P-Int and P-NC) (*P*<0.0001) (Fig. 4b). The microbiome has been shown to contribute high amounts of peptidoglycan in the host, acting as a potent immune factor [100], and it is involved in non-immune processes such as neurodevelopment and behaviour [101], as well as signalling among surrounding micro-organisms [102]. Bacterial cell wall biosynthesis is closely coordinated with cell division. Recently, it was reported that the inhibition of peptidoglycan synthesis has immediate drastic effects on *S. aureus* cell division [103], so a higher abundance in the presence of the peptidoglycan biosynthesis pathway in the nasal microbiome compared to the pharyngeal microbiome was expected.

Furthermore, this study reports a higher sequence abundance in the prediction of chondroitin sulphate I degradation (Fig. 4c), in the three pharyngeal microbiomes analysed (P-Persist, P-Int and P-NC) compared to the nasal microbiomes (N-Persist, N-Int and N-NC) (*P*<0.0001). Chondroitin sulphate is a glycosaminoglycan present in the extracellular matrix, participating in its maintenance and protection of the extracellular microenvironment. It also plays roles as a multifunctional and regulatory signal molecule, directly and indirectly affecting cell signalling in various physiological activities such as antioxidant, anti-inflammatory, anticoagulant and immunoregulatory [104]. *S. pneumoniae* can degrade hyaluronan and chondroitin sulphate [105], and as the genus *Streptococcus* is very abundant in the pharynx (Fig. 2b, Table 3), it was expected to be found in higher abundance in the pharyngeal microbiome compared to the nasal microbiome (Fig. 2b).

Regarding the three metabolic pathways that presented the most significant *P*-values, Jo *et al*. [106] report that *S. epidermidis* is capable of modifying the nasal intracellular environment that lacks polyamines and promotes the balance of cellular polyamines, restricting the replication of viruses such as influenza. Besides, polyamines have been reported to restrict the growth of *S. aureus* [107], and as seen in Fig. 4(d) N-NC is the only group of nasal carriers where an abundant presence of the polyamine biosynthesis pathway is found. The second route with a high significance *P*-value is the biosynthesis of l-aspartate and l-asparagine (Fig. 4e); a greater presence was found in the microbiomes of pharyngeal carriers compared to the microbiomes of nasal carriers (*P*<0.0001). Yang *et al*. [108] found that concentrations greater than 10 mM of aspartate inhibit the development of planktonic cells of *S. aureus*, thus decreasing the formation of biofilms, necessary to increase antibiotic resistance and persistence in the host. In this sense, we found that this pathway is more present in N-NC, compared to N-Persist (*P*<0.05). On the other hand, it has been reported that the inactivation of the Krebs cycle in *S. aureus* strains with mutations in *sucA* and *sucB* genes, which encode subunits of the enzyme *α*-ketoglutarate dehydrogenase, increases the survival of *S. aureus* at lethal concentrations of ciprofloxacin [109]. In this sense, Hobbs *et al*. [110], infected *Drosophila melanogaster* specimens with mutant strains of *S. aureus* of the Krebs cycle enzymes, finding that it increased the mortality of flies of both sexes. Therefore, these results suggest that the decrease in the Krebs cycle in *S. aureus* favours tolerance to mechanisms associated with the innate immunity of the host, benefiting the persistence of this bacteria. Fig. 4(f) show that TCA is present in a higher proportion in the microbiomes of nasal carriers compared to pharyngeal carriers (*P*<0.0001), but it is not possible to identify specific differences between the microbiomes of nasal carriers (N-Persist, N-Int and N-NC) or between the microbiomes of pharyngeal carriers (P-Persist, P-Int and P-NC). Therefore, all these processes will have to be studied further.

In conclusion, *S. aureus* can colonize and persist in the pharynx and nose; however, the incidence and persistence of *S. aureus* in the pharynx may be higher than in the nose. The nose microbiome exhibits higher bacterial diversity compared to the pharyngeal microbiome. The main difference lies in the composition of the microbiome according to the anatomical site analysed rather than the type of *S. aureus* carrier. These results suggest that the pharynx is also an important site of colonization of *S. aureus*, even though it is found in low abundance percentages and not only as a pathogenic species. No statistically significant differences were found when comparing the metataxonomy of the microbiome of the three types of *S. aureus* carriers in both the pharynx and nose among taxa with relative abundances greater than 1%; the most relevant differences may be in those organisms found in very low abundance. Therefore, the microbiome apparently does not influence the persistence of *S. aureus*.

## supplementary material

10.1099/jmm.0.001940 Uncited Supplementary Material 1.

## Abbreviations

KEGG: Kyoto Encyclopedia of Genes and Genomes
OTUs: operational taxonomic units
PCA: principal component analysis
SP: sample pool
SREC-1: scavenger receptor-1
TCA: tricarboxylic acid
TSB: trypticasein soy broth
WTA: wall teichoic acid
