# Supplementary material for: Comparison of the bacterial microbiome in the pharynx and nasal cavity of persistent, intermittent carriers and non-carriers of Staphylococcus aureus
Source: J Med Microbiol. 2024 Dec 4;73(12):001940. doi: 10.1099/jmm.0.001940 (PMC11616445; doi:10.1099/jmm.0.001940)
Supplement: Uncited Supplementary Material 1. [file jmm-73-01940-s001.pdf]

**Table S1**. Formation of sample pools from different groups of *Staphylococcus aureus* carriers in the pharynx and nose.

<table>
<tr><th>S. aureus carrier type</th><th>Carriers (N= 98)</th><th>Samples for pharynx pool</th><th>Samples for nose pool</th></tr>
<tr><td>1. Persistent pharynx and nose</td><td>18</td><td colspan="2">6 – 6 – 6</td></tr>
<tr><td>2. Persistente exclusive pharynx</td><td>19</td><td>6 – 6 – 7</td><td></td></tr>
<tr><td>3. No carriers</td><td>6</td><td colspan="2">2 – 2 – 2</td></tr>
<tr><td>4. Intermittent pharynx and nose</td><td>15</td><td colspan="2">5 – 5 – 5</td></tr>
<tr><td>5. Persistent nose, intermittent pharynx</td><td>5</td><td colspan="2">2 – 2 – 1</td></tr>
<tr><td>6. Persistent pharynx, intermittent nose</td><td>15</td><td colspan="2">5 – 5 – 5</td></tr>
<tr><td>7. Intermittent exclusive pharynx</td><td>20</td><td>7 – 7 – 6</td><td></td></tr>
</table>

**Table S2**. Metabolic pathways with statistical significance from functional analysis of pharyngeal and nasal microbiomes of *S. aureus* carriers and non-carriers.

| Pathway code | Pathway | *P*-values | P-Persist | P-Int | P-NC | N-Persist | N-Int | N-NC |
|---|---|---|---|---|---|---|---|---|
| | | | Percentages of relative abundance | | | | | |
| PWY-6565 | Polyamine biosynthesis III | 5.18E-19 | 0.00E+00 | 0.00E+00 | 0.00E+00 | 0.00E+00 | 4.49E-05 | 8.22E-04 |
| ASPASN-PWY | L-aspartate and L-asparagine biosynthesis | 1.26E-12 | 5.67E-01 | 5.54E-01 | 5.76E-01 | 6.00E-02 | 1.52E-01 | 2.28E-01 |
| PWY-5347 | L-methionine biosynthesis (transsulfuration) | 6.80E-11 | 5.77E-01 | 5.54E-01 | 5.90E-01 | 1.02E-01 | 2.08E-01 | 3.00E-01 |
| PWY-7254 | TCA cycle VII (acetate-producers) | 1.95E-10 | 2.43E-01 | 2.34E-01 | 1.99E-01 | 7.88E-01 | 7.03E-01 | 5.68E-01 |
| TCA | TCA cycle | 1.91E-09 | 3.40E-01 | 3.29E-01 | 2.87E-01 | 8.73E-01 | 7.83E-01 | 7.04E-01 |
| P4-PWY | L-lysine, L-threonine and L-methionine biosynthesis I | 2.03E-09 | 5.07E-01 | 4.97E-01 | 5.28E-01 | 1.53E-01 | 2.53E-01 | 3.81E-01 |
| REDCITCYC | TCA cycle VI (Helicobacter) | 8.24E-09 | 2.45E-01 | 2.19E-01 | 1.84E-01 | 8.10E-01 | 7.35E-01 | 5.80E-01 |
| PWY-7013 | (*S*)-propane-1,2-diol degradation | 1.43E-08 | 2.80E-01 | 2.62E-01 | 3.06E-01 | 3.38E-03 | 1.55E-02 | 6.89E-03 |
| NONOXIPENT-PWY | Pentose phosphate pathway (non-oxidative branch) I | 3.96E-08 | 8.81E-01 | 8.77E-01 | 8.73E-01 | 6.15E-01 | 6.49E-01 | 5.86E-01 |
| PWY-7377 | Cob(II)yrinate *a,c*-diamide biosynthesis I (early cobalt insertion) | 5.22E-08 | 2.75E-01 | 2.72E-01 | 2.49E-01 | 5.88E-03 | 2.18E-02 | 3.31E-02 |
| PANTO-PWY | Phosphopantothenate biosynthesis I | 5.28E-07 | 4.43E-01 | 4.07E-01 | 3.82E-01 | 7.04E-01 | 6.10E-01 | 5.84E-01 |
| THISYN-PWY | Superpathway of thiamine diphosphate biosynthesis I | 1.00E-06 | 4.36E-01 | 4.27E-01 | 4.21E-01 | 8.84E-02 | 1.72E-01 | 2.13E-01 |
| PWY-5265 | Peptidoglycan biosynthesis II (staphylococci) | 1.59E-06 | 8.61E-03 | 5.32E-03 | 1.02E-02 | 1.51E-01 | 1.54E-01 | 2.70E-01 |
| PWY-7003 | Glycerol degradation to butanol | 2.05E-06 | 1.11E-01 | 1.20E-01 | 1.62E-01 | 1.05E-02 | 2.29E-02 | 5.10E-02 |
| PWY-7111 | Pyruvate fermentation to isobutanol (engineered) | 2.14E-06 | 6.80E-01 | 7.00E-01 | 6.63E-01 | 1.02E+00 | 1.00E+00 | 1.03E+00 |
| SO4ASSIM-PWY | Assimilatory sulfate reduction I | 4.03E-06 | 2.21E-01 | 1.70E-01 | 1.32E-01 | 8.18E-01 | 5.73E-01 | 5.66E-01 |
| P108-PWY | Pyruvate fermentation to propanoate I | 4.16E-06 | 3.85E-01 | 3.63E-01 | 3.53E-01 | 5.03E-02 | 8.63E-02 | 2.13E-01 |
| PWY-6892 | Thiazole component of thiamine diphosphate biosynthesis I | 5.38E-06 | 2.51E-01 | 2.43E-01 | 2.52E-01 | 3.14E-02 | 6.88E-02 | 6.78E-02 |
| TRNA-CHARGING-PWY | Trna charging | 5.72E-06 | 6.97E-01 | 6.92E-01 | 7.08E-01 | 4.68E-01 | 5.12E-01 | 4.96E-01 |
| FUC-RHAMCAT-PWY | Superpathway of fucose and rhamnose degradation | 7.92E-06 | 3.41E-02 | 5.01E-02 | 3.97E-02 | 1.75E-03 | 7.41E-03 | 7.25E-03 |
| PWY-5104 | L-isoleucine biosynthesis IV | 1.31E-05 | 5.61E-01 | 5.82E-01 | 5.98E-01 | 7.40E-02 | 3.70E-01 | 2.07E-01 |
| PWY-6385 | Peptidoglycan biosynthesis III (mycobacteria) | 1.51E-05 | 6.90E-01 | 6.92E-01 | 7.08E-01 | 3.98E-01 | 5.02E-01 | 5.24E-01 |
| PWY-5677 | Succinate fermentation to butanoate | 2.99E-05 | 4.15E-02 | 4.09E-02 | 4.85E-02 | 8.27E-04 | 3.25E-03 | 5.81E-03 |
| SULFATE-CYS-PWY | Superpathway of sulfate assimilation and cysteine biosynthesis | 3.12E-05 | 2.99E-01 | 2.46E-01 | 1.90E-01 | 7.47E-01 | 5.55E-01 | 5.79E-01 |

| | | | | | | | | |
|---|---|---|---|---|---|---|---|---|
| KDO-NAGLIPASYN-PWY | Superpathway of $(Kdo)_2$-lipid A biosynthesis | 3.68E-05 | 1.97E-01 | 1.92E-01 | 2.83E-01 | 5.65E-03 | 2.54E-02 | 9.54E-03 |
| FUCCAT-PWY | L-fucose degradation I | 3.98E-05 | 6.94E-02 | 9.14E-02 | 1.40E-01 | 2.31E-03 | 1.04E-02 | 8.80E-03 |
| PWY-5741 | Ethylmalonyl-coa pathway | 4.35E-05 | 1.11E-05 | 0.00E+00 | 9.71E-06 | 1.15E-03 | 3.15E-03 | 9.31E-03 |
| GLUCARDEG-PWY | *D*-glucarate degradation I | 4.47E-05 | 1.77E-05 | 1.04E-03 | 4.40E-05 | 1.56E-03 | 2.76E-03 | 7.65E-03 |
| GLUCARDEG-PWY | D-galactarate degradation I | 4.47E-05 | 1.77E-05 | 1.04E-03 | 4.40E-05 | 1.56E-03 | 2.76E-03 | 7.65E-03 |
| GLUCARGALACTSUPER-PWY | Superpathway of D-glucarate and D-galactarate degradation | 4.48E-05 | 1.77E-05 | 1.03E-03 | 4.40E-05 | 1.56E-03 | 2.78E-03 | 7.64E-03 |
| PWY-4984 | Urea cycle | 5.70E-05 | 4.34E-02 | 2.30E-02 | 6.79E-03 | 1.46E-01 | 2.03E-01 | 3.68E-01 |
| PWY-6182 | Superpathway of salicylate degradation | 9.04E-05 | 6.26E-04 | 1.56E-03 | 1.69E-03 | 3.20E-02 | 7.40E-02 | 1.23E-01 |
| PWY-6749 | CMP-legionaminate biosynthesis I | 2.16E-04 | 1.40E-01 | 1.36E-01 | 9.14E-02 | 2.91E-04 | 3.83E-03 | 3.40E-03 |
| PWY-1501 | Mandelate degradation I | 5.53E-04 | 0.00E+00 | 0.00E+00 | 0.00E+00 | 4.68E-04 | 8.19E-04 | 3.08E-03 |
| MET-SAM-PWY | Superpathway of *S*-adenosyl-L-methionine biosynthesis | 5.62E-04 | 5.53E-01 | 5.38E-01 | 5.76E-01 | 2.62E-01 | 3.91E-01 | 3.87E-01 |
| PWY-6143 | CMP-pseudaminate biosynthesis | 6.57E-04 | 4.56E-03 | 5.92E-03 | 5.45E-03 | 0.00E+00 | 6.71E-05 | 0.00E+00 |
| SER-GLYSYN-PWY | Superpathway of L-serine and glycine biosynthesis I | 1.30E-03 | 5.29E-01 | 4.85E-01 | 4.95E-01 | 7.06E-01 | 6.71E-01 | 6.19E-01 |
| HOMOSER-METSYN-PWY | L-methionine biosynthesis I | 1.42E-03 | 4.62E-01 | 4.41E-01 | 4.73E-01 | 1.89E-01 | 3.29E-01 | 2.87E-01 |
| PWY0-1277 | Pathway: 3-phenylpropanoate and 3-(3-hydroxyphenyl)propanoate degradation | 1.51E-03 | 2.86E-03 | 2.41E-03 | 1.69E-04 | 3.02E-03 | 5.79E-03 | 2.05E-02 |
| PWY-7031 | Protein *N*-glycosylation (bacterial) | 2.12E-03 | 6.37E-03 | 8.10E-03 | 7.38E-03 | 4.68E-04 | 1.11E-04 | 3.82E-04 |
| PWY-5097 | L-lysine biosynthesis VI | 2.23E-03 | 6.22E-01 | 6.18E-01 | 6.38E-01 | 3.82E-01 | 5.05E-01 | 5.40E-01 |
| PWY-5705 | Allantoin degradation to glyoxylate III | 2.64E-03 | 1.27E-03 | 1.24E-02 | 2.04E-03 | 3.16E-02 | 2.58E-02 | 7.05E-02 |
| TEICHOICACID-PWY | Poly(glycerol phosphate) wall teichoic acid biosynthesis | 2.85E-03 | 1.93E-02 | 3.36E-02 | 1.95E-02 | 3.89E-01 | 1.79E-01 | 2.11E-01 |
| PWY-6572 | Chondroitin sulfate degradation I (bacterial) | 3.23E-03 | 7.50E-03 | 6.01E-03 | 6.20E-03 | 0.00E+00 | 6.04E-05 | 1.23E-04 |
| PWY-7373 | Superpathway of demethylmenaquinol-6 biosynthesis II | 4.65E-03 | 6.36E-03 | 7.10E-03 | 6.99E-03 | 4.80E-04 | 1.01E-04 | 7.64E-04 |
| FAO-PWY | Fatty acid β-oxidation I (generic) | 5.31E-03 | 1.72E-01 | 1.01E-01 | 7.84E-02 | 5.15E-01 | 6.97E-01 | 6.17E-01 |
| PWY-7371 | 1,4-dihydroxy-6-naphthoate biosynthesis II | 8.86E-03 | 7.41E-03 | 7.82E-03 | 9.31E-03 | 5.39E-04 | 4.44E-04 | 1.62E-03 |
| PWY-5861 | Superpathway of demethylmenaquinol-8 biosynthesis I | 8.90E-03 | 3.48E-01 | 3.51E-01 | 3.75E-01 | 1.85E-01 | 1.49E-01 | 2.19E-01 |
| PWY-6185 | 4-methylcatechol degradation (*ortho* cleavage) | 2.62E-02 | 3.33E-04 | 2.69E-04 | 9.18E-04 | 1.99E-02 | 5.75E-02 | 5.98E-02 |
| AEROBACTINSYN-PWY | Aerobactin biosynthesis | 2.70E-02 | 6.36E-06 | 0.00E+00 | 0.00E+00 | 2.08E-04 | 1.14E-03 | 1.03E-03 |
| PWY-7046 | 4-coumarate degradation (anaerobic) | 2.77E-02 | 0.00E+00 | 0.00E+00 | 0.00E+00 | 2.30E-04 | 9.75E-04 | 1.35E-03 |
| PWY-6353 | Purine nucleotides degradation II (aerobic) | 2.88E-02 | 1.05E-01 | 1.29E-01 | 5.45E-02 | 1.93E-01 | 2.33E-01 | 4.13E-01 |

| | | | | | | | | |
|---|---|---|---|---|---|---|---|---|
| METHYLGALLATE-DEGRADATION-PWY | Methylgallate degradation | 3.13E-02 | 1.19E-03 | 1.46E-02 | 8.93E-04 | 3.07E-02 | 1.57E-01 | 9.87E-02 |
| PWY-5838 | Superpathway of menaquinol-8 biosynthesis I | 3.14E-02 | 3.78E-01 | 3.79E-01 | 4.03E-01 | 2.31E-01 | 1.83E-01 | 2.69E-01 |
| CALVIN-PWY | Calvin-Benson-Bassham cycle | 4.01E-02 | 7.39E-01 | 7.43E-01 | 7.62E-01 | 6.64E-01 | 6.06E-01 | 5.98E-01 |
| PWY-6969 | TCA cycle V (2-oxoglutarate synthase) | 4.69E-02 | 3.07E-01 | 3.46E-01 | 2.92E-01 | 5.79E-01 | 5.28E-01 | 6.05E-01 |
| PWY-3801 | Sucrose degradation II (sucrose synthase) | 4.81E-02 | 0.00E+00 | 0.00E+00 | 0.00E+00 | 3.33E-03 | 2.07E-03 | 1.08E-02 |
| GALLATE-DEGRADATION-I-PWY | Gallate degradation II | 4.91E-02 | 9.53E-04 | 1.20E-02 | 7.15E-04 | 2.52E-02 | 1.48E-01 | 8.46E-02 |

P-Persist: persistent carrier in pharynx, P-Int: intermittent carrier in pharynx, P-NC: non-carrier in pharynx, N-Persist: persistent carrier in nose, N-Int: intermittent carrier in nose and N-NC: non-carrier in nose.

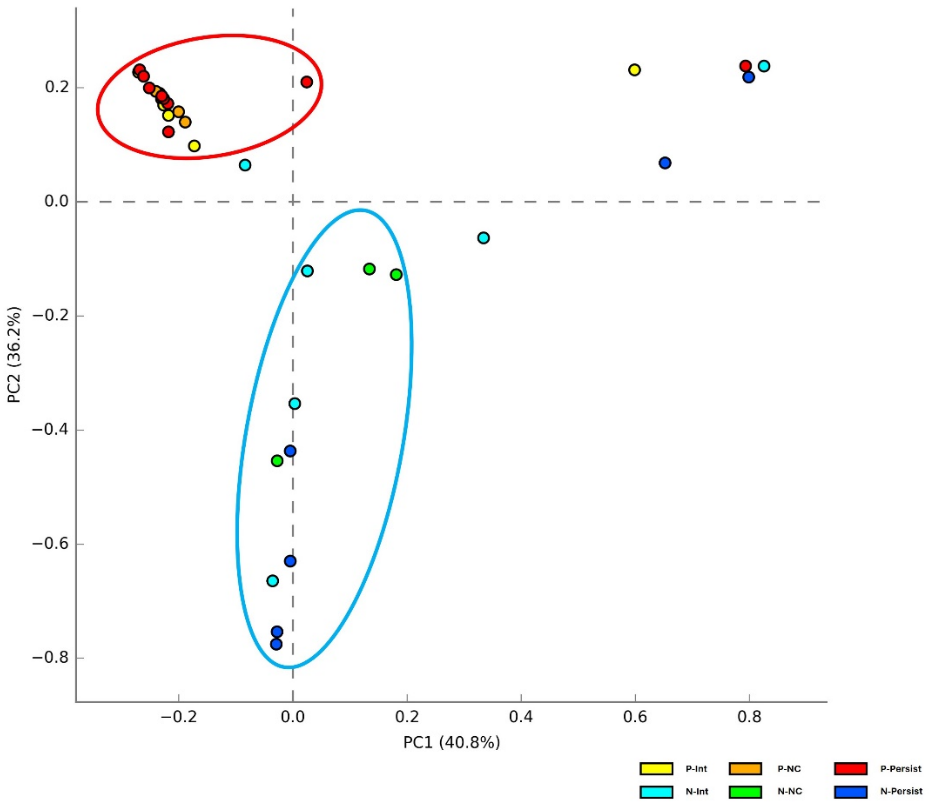


**Supplementary Figure 1**. Principal component analysis (PCA) plot of SP of *S. aureus* carrier types in pharynx and nose at bacterial order level. SP: sample pools, P-Persist: persistent carrier in pharynx, P-Int: intermittent carrier in pharynx, P-NC: non-carrier in pharynx, N-Persist: persistent carrier in nose, N-Int: intermittent carrier in nose and N-NC: non-carrier in nose.

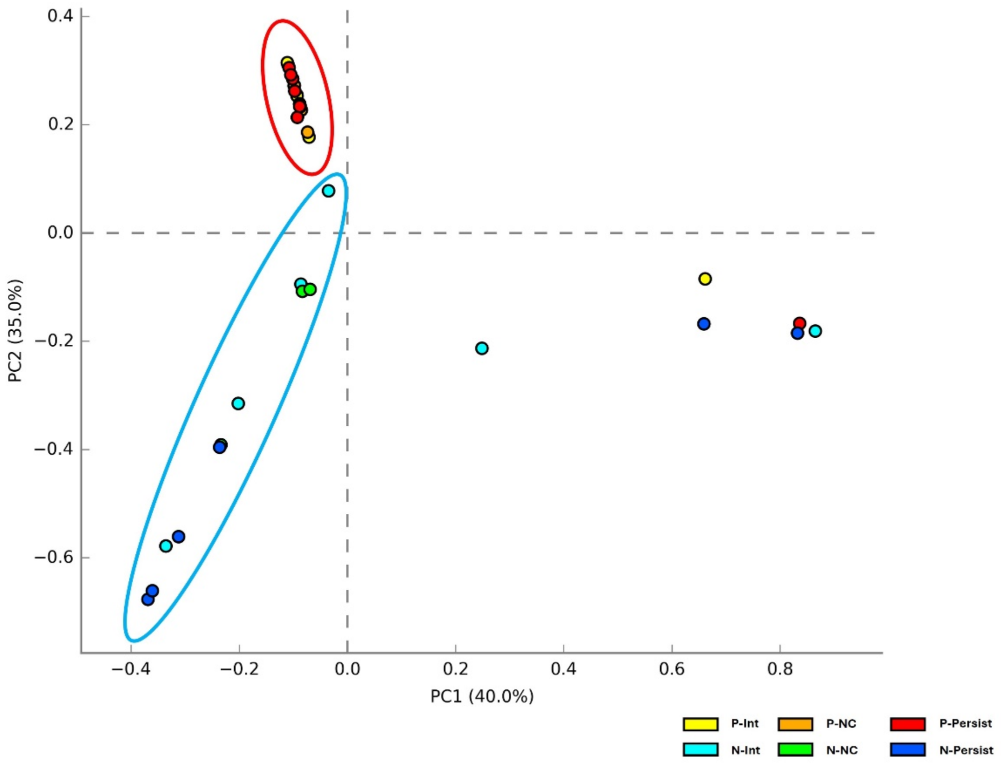


**Supplementary Figure 2**. Principal component analysis (PCA) plot of SP of *S. aureus* carrier types in pharynx and nose at bacterial family level. SP: sample pools, P-Persist: persistent carrier in pharynx, P-Int: intermittent carrier in pharynx, P-NC: non-carrier in pharynx, N-Persist: persistent carrier in nose, N-Int: intermittent carrier in nose and N-NC: non-carrier in nose.
